# *TP53BP2* Promotes Placental Autophagy and Preeclampsia via G9a and *DNMT1* Cooperatively Modulating E2F1

**DOI:** 10.1002/advs.202516408

**Published:** 2026-01-07

**Authors:** Nan Jiang, Shaoju Jin, Shaoying Wen, Wen Zeng, Chen Wang, Jingyu Wang, Qingyun Song, Guizhong Li, Pengzhi Yin, Yuhui Liao, Yuee Chai, Huiping Zhang, Shengchao Ma

**Affiliations:** ^1^ School of Basic Medical Science Central South University Changsha China; ^2^ LuoHe Medical College LuoHe China; ^3^ NHC Key Laboratory of Metabolic Cardiovascular Diseases Research Ningxia Medical University Yinchuan China; ^4^ Key Laboratory of Vascular Injury and Repair Research of the Ningxia the Ningxia Hui Autonomous Region Ningxia Medical University Yinchuan China; ^5^ Department of Scientific Research and Teaching The Central Hospital of Shaoyang City Shaoyang China; ^6^ Xiangya Medical College Central South University Changsha China; ^7^ College of Life Sciences Central South University Changsha China; ^8^ Faculty of Biomedical Engineering The Chinese University of Hong Kong Hong Kong China; ^9^ Institute For Engineering Medicine Kunming Medical University Kunming China; ^10^ State Key Laboratory of Functions and Applications of Medicinal Plants Guizhou Provincial Engineering Technology Research Center For Chemical Drug R&D Guiyang China; ^11^ Department of Medical Genetics Maternal and Child Health Hospital of Hunan Province Changsha China

**Keywords:** autophagy, DNA methylation, histone methylation, preeclampsia, tumor suppressor p53‐binding protein 2

## Abstract

Preeclampsia (PE) is a pregnancy‐related disorder characterized by impaired migration and invasion of trophoblast cells. Recent studies have highlighted the critical role of autophagy in the development of PE. However, the precise mechanisms underlying the upregulation of autophagy in PE remain unclear. This study demonstrated that the expression of the tumor suppressor p53‐binding protein 2 (TP53BP2) is significantly upregulated in patients with PE. Silencing of *TP53BP2* not only decreases autophagy but also attenuates PE progression in rat model. Moreover, *TP53BP2* expression was positively correlated with blood pressure and body mass index (BMI) but negatively correlated with gestational age at delivery and neonatal birth weight. Our findings suggest that *TP53BP2* enhances autophagy by promoting the release of Beclin‐1 from the Bcl‐2/Beclin‐1 complex. Additionally, DNMT1 and G9a cooperatively downregulated *TP53BP2* expression by reducing DNA methylation and H3K9me2 enrichment in the *TP53BP2* promoter region. Importantly, the cooperation between DNMT1 and G9a suppressed E2F1 binding to the *TP53BP2* promoter, leading to transcriptional repression of *TP53BP2* in trophoblasts. In brief, our study indicates that *TP53BP2* promotes autophagy in trophoblasts through DNA methylation and H3K9me2‐mediated transcriptional regulation. These findings suggest that targeting *TP53BP2* may be a potential therapeutic strategy for PE.

## Graphical abstract

G9a, and DNA Methyltransferase1 (DNMT1) cooperatively modulates E2F1 on the promoter of tumor suppressor p53‐binding protein 2 (TP53BP2) increased autophagy in preeclampsia. TP53BP2 promotes autophagy in trophoblasts through DNA methylation and H3K9me2‐mediated transcriptional regulation.

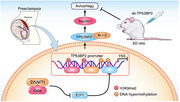

## 1 Introduction

Preeclampsia (PE), which affects 3%–7% of all pregnancies, is a leading cause of maternal and fetal morbidity and mortality worldwide [1]. PE can be categorized into two primary types based on the timing of onset: early and late. Early onset PE, which typically occurs before 34 weeks of gestation, is a severe condition characterized by elevated blood pressure (BP) and often significant proteinuria [2]. This form of PE is particularly concerning because it cannot always be prevented [3]. Insufficient trophoblast invasion, impaired uterine spiral artery remodeling, placental dysfunction, and endothelial dysfunction have all been reported in early‐onset PE pregnancies [4]. Autophagy is a fundamental biological process that facilitates cellular degradation and the recycling of components. During early pregnancy, autophagy plays a crucial role in embryogenesis and is essential for normal embryonic development [5, 6]. However, under oxygen deprivation conditions, autophagy can accelerate trophoblast aging, exacerbating trophoblast dysfunction and deficiency [7]. Clinically, increased LC3B‐mediated autophagy has been implicated in the pathogenesis of PE [8]. Additionally, previous studies have shown that autophagy is upregulated in the placentas of PE pregnancies and in trophoblasts under hypoxic conditions, suggesting that inhibiting autophagy may be a potential therapeutic strategy for PE [9]. Despite these findings, the precise role of autophagy in PE trophoblasts remains unclear, and the initial factors that trigger this process are still not well understood.

The tumor suppressor p53‐binding protein 2 (*TP53BP2*) gene encodes a protein that plays a major role in regulating apoptosis [10]. Endogenous *TP53BP2* is damage‐inducible and modulates physiologic damage response pathways involved in diverse cellular functions [11]. Recent studies have reported that *TP53BP2* is overexpressed in various tumors and is a critical factor in tumorigenesis and tumor development [12, 13]. Additionally, *TP53BP2* significantly influences the proliferation and metastasis of triple‐negative breast cancer cells, with its functional mechanisms being largely p53‐independent [14].Recent findings have shown that *TP53BP2* can regulate autophagy through its N‐terminal domain, which shares high structural similarity with *ATG12* and *LC313* due to the ubiquitin‐fold sharing motif [15]. Liu et al. reported that *TP53BP2* overexpression inhibited autophagy at low‐dose gp120 (a soluble envelope glycoprotein; 50 ng/mL) but induced autophagy at high‐dose gp120 (200 ng/mL) in SH‐SY5Y neuroblastoma cells; conversely, *TP53BP2* knockdown attenuated autophagy induced by high‐dose gp120 [16]. These results suggest that *TP53BP2* regulates autophagy in a gp120 concentration‐dependent manner. Moreover, studies have shown that *TP53BP2* inhibits RAS‐induced autophagic activity to dictate the cellular response to RAS [17]. Given that impaired trophoblast invasion and incomplete spiral artery remodeling are among the main causes of PE [18, 19], the aberrant expression and dual function of *TP53BP2* led us to hypothesize that this molecule may be involved in PE pathogenesis through the regulation of autophagy.

Abnormal DNA methylation during placentation is the most important epigenetic factor associated with PE [20]. Changes in histone modifications, such as acetylation, can also result in the development of PE. Gene expression is regulated by various factors, such as DNA methylation marks or binding sites for transcription factors [21]. Among histone modifications, histone methylation is a complex epigenetic mechanism that can activate or repress target gene transcription by altering the chromosomal structure, depending on the location of the methylation site [22]. Interestingly, DNA methylation regulates gene expression by suppressing gene transcription. Similarly, DNA methylation modifies chromatin structure and interacts with other epigenetic modifications, thereby enabling a more diverse regulation of gene expression [23, 24]. In mammals, DNA methylation patterns in somatic cells are primarily determined by *DNMT1* activity [25]. The direct interaction between *DNMT1* and G9a is proposed to coordinate DNA methylation and H3K9 methylation during DNA replication [26]. The transcription activity of a specific gene is regulated by epigenetic markers and the interplay between transcription factors and the cis‐elements of specific promoters in a time‐ and space‐dependent manner, which is intricately linked to gene expression [27]. Additionally, *E2F1*, a member of the E2F family, is involved in the regulation of cell cycle progression, cell differentiation, and DNA repair [28, 29]. Evidence suggests that the specific binding of *E2F1* and/or *E2F2* to CpG islands protects against *de novo* DNA methylation through nucleosome depletion [30]. Moreover, increased *E2F1* expression and CpG hydroxymethylation of the *E2F1* binding motif conjointly induce *ESRP1* expression in breast cancer [31]. Reports have suggested that searching for abnormal DNA (hypo/hyper)‐methylation could be a sensible approach to discovering new markers related to PE, aiming to predict and understand PE development [32].

## 2 Methods and Materials

### 2.1 Patients and Study Samples

Eighty‐five placentas were obtained from women who underwent cesarean delivery at the General Hospital of Ningxia Medical University between 2017 and 2021. PE was defined as systolic BP (SBP) of ≥140 mmHg and diastolic BP (DBP) of ≥90 mmHg in two consecutive measurements obtained at least 6 h apart, along with proteinuria (≥0.3 g/24 h) after 20 weeks of gestation. Placentas from non‐PE pregnant volunteers (pregnant controls; n = 40) with no medical history or medication use served as controls. Placentas from the PE pregnancy group (n = 45) were obtained from pregnant patients who delivered with early onset PE (<34 weeks of gestation). The exclusion criteria were multiple gestations, fetal congenital malformations or chromosomal abnormalities, recent infection, antiphospholipid antibodies, trauma, drug or alcohol abuse during pregnancy, hypertension before 20 weeks of gestation, thrombophilia with a history of PE, history of anticoagulant/antiaggregation therapy, smoking, and incomplete data from obstetric examinations. The central part of the placenta was collected within 10 min of cesarean delivery, avoiding macroscopic areas of infarction and calcification. After rinsing briefly with saline, the samples were frozen at −80°C or fixed in polyformaldehyde until further analysis. All experiments were conducted following the protocol approved by the Clinical Research Ethics Committee of Ningxia Medical University (NO. 2017–083). Informed consent was obtained from all the patients.

### 2.2 Animal Experiments

All animal experiments were performed using Sprague–Dawley (SD) rats (13–14 weeks) housed at the Ningxia Medical University Laboratory Animal Center (Yinchuan, China). The rats were housed in a temperature‐controlled room (22°C–24°C) with a 12‐h light/dark cycle and had free access to food and water. After 1 week of acclimatization, the rats were mated with healthy male SD rats at a 2:1 ratio. The onset of gestation was identified by vaginal sperm plugs on gestational day (GD) 1. The most reliable animal model of PE was the surgically induced reduced uterine perfusion pressure (RUPP) model. This model induces hypertension, proteinuria, renal dysfunction, antiangiogenic state, inflammation, oxidative stress, cardiac dysfunction, and intrauterine growth restriction, similar to PE in humans. Thus, on GD14, the rats underwent surgical RUPP under pentobarbital anesthesia. Briefly, a midline incision was made to open the abdominal cavity and expose the lower abdominal aorta. Silver clips (0.203 mm) were placed around the aorta above the iliac bifurcation to the RUPP by approximately 40%. Simultaneously, silver clips (0.100 mm) were used to decrease the degree of ovarian collateral circulation in the bilateral uterine arteries at the ovarian ends of the uterine arch. The sham group underwent the same procedure as the RUPP group but without clip placement. Recombinant adeno‐associated virus (AAV) serotype 9 vectors carrying *TP53BP2* short hairpin RNA (shRNA; AAV‐*shTP53BP2*), *DNMT1* shRNA (AAV‐*DNMT1*), and G9a shRNA (AAV‐*shG9a*), or recombinant AAV9 vectors carrying a negative control (AAV‐shNC) were manufactured by GeneChem Inc. (Shanghai, China). Subsequently, virus (10 µL, 1.5E+11 particles) was injected into the placenta. On GD20, the rats were euthanized using pentobarbital, and the pups were removed and weighed. The placentas were washed with ice‐cold physiological salt solution and preserved at −80°C for subsequent analysis. The animal experiments were approved by the Committee on the Ethics of Animal Experiments of Ningxia Medical University (NO. 2021–250).

### 2.3 Cell Culture and Treatment

HTR‐8/SVneo (RRID: CVCL_7162), and JEG‐3 (RRID: CVCL_0363) cells were obtained from the American Type Culture Collection. To ensure the absence of mycoplasma contamination, the cells were characterized as mycoplasma‐negative using the MycoBlue Mycoplasma Detector (D101‐02;Vazyme, Nanjing, Jiangsu, China) following the manufacturer's instructions. The cells were maintained in either RPMI‐1640 or Ham's F‐12 medium enriched with 10% fetal bovine serum (FBS) and an antibiotic–antimycotic solution [100 U/mL penicillin (Solarbio,Beijing,China) or 100 mg/mL streptomycin (Solarbio,China)] under a 5% CO_2_ humidified atmosphere at 37°C. To simulate severe hypoxia, cells were plated at a density of 60 mm. After 24 h, the cells were placed in a NAPCO Series 8000WJ incubator (Thermo Fisher Scientific, Waltham, MA, USA) under 1% O_2_ and 5% CO_2_ at 37°C for 48 h. HEK293T cells were cultured in Dulbecco's modified Eagle's medium supplemented with 10% FBS, 100 U/mL penicillin, and 100 mg/mL streptomycin. For the transfection experiments, cells were subjected to viral infection with recombinant adenoviruses encoding *TP53BP2* (Ad‐*TP53BP2*), DNMT1 (Ad‐*DNMT1*), *E2F1* (Ad‐*E2F1*), and *G9a* (Ad‐*G9a*). Adenoviruses encoding green fluorescent protein (GFP) were used as a negative controls (Ad‐NC). After 4 h of incubation, the culture medium was replaced with regular RPMI‐1640 supplemented with 7% FBS, and the cells were subjected to hypoxic conditions at 37°C with 5% CO_2_ for 48 h. Additionally, these two cell lines were transfected with lentiviruses carrying sh‐*TP53BP2*, sh‐Beclin‐1, sh‐*DNMT1*, sh‐*E2F1*, or sh‐*G9a*, or sh‐NC using Lipofectamine 2000 (Thermo Fisher Scientific, USA), according to the manufacturer's protocol. After 6 h of incubation, the cells were cultured in 7% FBS under hypoxic conditions at 37°C with 5% CO_2_ for 48 h.

### 2.4 RNA‐Sequencing (RNA‐seq) Assay

Total RNA was extracted from the placental tissues to construct cDNA libraries. For the small RNA cDNA library, the complete RNA was first ligated with an RNA 3′‐adapter and a 5′‐adapter. Subsequently, reverse transcription primers were used to convert the ligated RNAs into cDNAs. The resulting cDNAs were amplified using polymerase chain reaction (PCR) and purified by gel electrophoresis. The cDNA quality was assessed using an Agilent 2100 chip (Agilent, Santa Clara, CA, USA). For the analysis of the RNA‐seq library, total RNA was purified to eliminate rRNA with the Ribo‐Zero rRNA Removal Kit (Epicenter‐Illumina, Madison, WI, USA), followed by RNA fragmentation. Fagmented RNA was converted into first‐strand cDNA using a TruSeq Stranded Kit (Epicenter‐Illumina, San Diego, CA). Double‐stranded cDNA was generated via DNA polymerase I and RNase H. The 3′‐ends of the double‐stranded cDNA were adenylated and ligated with adapters. PCR amplification and purification were performed to construct a cDNA library. The libraries were sequenced using the Illumina HiSeq 2500 platform for total RNA‐seq, which uses a 90‐bp paired‐end sequencing strategy, whereas small RNA‐seq was performed using the Illumina HiSeq X Ten platform.

### 2.5 Transmission Electron Microscopy (TEM)

The placentas were cut into ≈1 × 1 × 1‐mm pieces. HTR‐8/SVneo and JEG‐3 cells were washed with ice‐cold phosphate‐buffered saline (PBS), digested with trypsin, and collected by centrifugation. Tissues and cells were subsequently fixed using a fixative buffer comprising 2% paraformaldehyde (PFA) and 2.5% glutaraldehyde in 0.1 M phosphate buffer. After fixation, the samples were embedded, sectioned to at a thickness of 0.12 µm and stained with uranyl acetate solution and lead citrate solution. The ultrathin sections were visualized using a JEOL TEM (Zeiss, Oberkochen, Germany).

### 2.6 Immunofluorescence Staining

Frozen sections of human and rat placentas were fixed in 4% PFA for 15 min and permeabilized with 0.2% Triton X‐100 for 15 min. After blocking with 10% goat serum, the sections were incubated overnight at 4°C using the following primary antibodies: TP53BP2 (Mouse, 1:500, Santa Cruz Biotechnology, Dallas, TX, USA, Cat#sc‐53861, RRID: AB_2206774), E2F1 (Mouse, 1:100, Santa Cruz Biotechnology, Texas, USA, Cat#sc‐251, RRID: AB_627476), G9a (Mouse, 1:500, Santa Cruz Biotechnology, Texas, USA, Cat#sc‐515726, RRID: AB_2942090), DNMT1 (Mouse, 1:500, Santa Cruz Biotechnology, Texas, USA, Cat#sc‐271729, RRID: AB_10710384), H3K9me2 (Mouse, 1:500, Abcam, Cambridge, UK, Cat# ab1220, RRID: AB_449854), CK‐7 (Rabbit, 1:50, Abcam, Cambridge, USA, Cat# ab181598, RRID: AB_2783822).The sections were subsequently washed thrice with PBS and incubated with fluorescein‐conjugated secondary antibodies (Goat, 1:500, Abcam, Cambridge, USA, Cat# ab7064, RRID: AB_955234; Goat, 1:500, Abcam, Cambridge, USA, Cat# ab150077, RRID: AB_2630356) for 1 h at 37°C. To visualize the cell nuclei, the sections were stained with 4′,6‐diamidino‐2‐phenylindole (DAPI). Subsequently, fluorescence was observed and imaged using laser confocal microscopy (Zeiss, Jena, Germany).

### 2.7 Immunohistochemistry (IHC)

Human and rat placental tissue sections were deparaffinized in xylene and rehydrated through a graded series of ethanol solutions. Antigen retrieval was performed by immersing the sections in 10‐mM citrate buffer (pH = 6.0) and heating them in a pressure cooker for 15 min. Endogenous peroxidase activity was quenched by incubating the sections in 3% hydrogen peroxide solution for 10 min. Subsequently, the sections were blocked with 5% inactivated goat serum for 1 h at 26°C and incubated overnight at 4°C with the following primary antibodies: *TP53BP2*(Rabbit, 1:500, ABclonal, Wuhan, China, Cat# A5704, RRID: AB_2766463), *LC3B* (Rabbit, 1:200, Abcam, Cambridge, USA, Cat# ab192890, RRID: AB_2827794), *p62* (Rabbit, 1:200, Abcam, Cambridge, USA, Cat# ab109012, RRID: AB_2810880).After washing the sections with PBS, they were incubated with the secondary antibody for 2 h at room temperature. Immunoreactivity was visualized by adding the 3,3′‐diaminobenzidine substrate solution. The sections were counterstained with Harris hematoxylin for 30 s to visualize cell nuclei. Finally, the sections were dehydrated using ethanol and xylene, and images were captured via an optical microscope(Olympus,Olympus Corporation,Japan).

### 2.8 Autophagic Flux Assay

GFP‐RFP‐LC3 adenoviruses (Hanbio, Shanghai, China) were used to monitor the autophagic flux. Briefly, when the cells reached 60% confluence, GFP‐RFP‐LC3 adenoviruses were added to the culture medium. After 8 h of adenoviral infection, the culture medium was replaced, and the cells were further incubated for 24 h. Cells were then fixed with 4% formaldehyde for 10 min. Fluorescence images were captured using a laser confocal microscope (Zeiss, Jena, Germany). Typically, an increased number of red puncta (representing autophagic lysosomes) compared to yellow puncta (formed by the overlap of red and green) indicates activated autophagy, whereas a greater number of yellow puncta relative to red puncta suggests autophagy suppression.

### 2.9 Western Blotting

Tissues and cells were lysed in ice‐cold lysis buffer (KeyGEN Biotech, Nanjing, China) and centrifuged at 4°C for 15 min. Cell lysates were separated by 8% sodium dodecyl sulfate‐polyacrylamide gel electrophoresis and transferred on to polyvinylidene difluoride membranes (MilliporeSigma, Burlington, MA, USA). After blocking with 5% nonfat milk, the membranes were incubated overnight at 4°C with specific primary antibodies against the following proteins: *TP53BP2* (Rabbit, 1:1000, ABclonal, Wuhan, China, Cat# A15105, RRID: RRID: AB_2761989), *LC3B* (Rabbit, 1:1000, Abcam, Cambridge, USA, Cat# ab192890, RRID: AB_2827794), *p62* (Rabbit, 1:1000, Abcam, Cambridge, USA, Cat# ab109012, RRID: AB_2810880), *Beclin‐1* (Rabbit, 1:1000, Abcam, Cambridge, USA, Cat# ab210498, RRID: AB_2810879), *Bcl‐2* (Rabbit, 1:1000, Abcam, Cambridge, USA, Cat# ab32124, RRID: AB_725644), *E2F1* (Rabbit, 1:1000, Abcam, Cambridge, USA, Cat# ab288369, RRID: AB_ 3086690), *DNMT1* (Rabbit, 1:1000, Abcam, Cambridge, USA, Cat# ab188453, RRID: AB_2877711), and *G9a* (Mouse, 1:500, Santa Cruz Biotechnology, Texas, USA, Cat#sc‐515726, RRID: AB_2942090).After three washes, the membranes were incubated with horseradish peroxidase‐conjugated secondary antibodies for 1 h, and protein expression was detected using a chemiluminescence kit (KeyGEN, Nanjing, China). The optical density of each band was analyzed via densitometry and normalized to that of a β‐actin loading control.

### 2.10 Quantitative Real‐Time PCR (qRT‐PCR) Analysis

Total RNA was isolated from human placentas and cells using an RNA isolation kit (TIANGEN BioTech, Beijing, China) according to the manufacturer's instructions. After RNA quantification, cDNA was synthesized using a reverse transcription kit (Takara Bio Inc., Shiga, Japan). cDNA was subsequently subjected to RT‐PCR amplification in a thermal cycler using the TBGreen Fast qPCR mix (Takara Bio Inc., Shiga, Japan). All experiments were performed in triplicate, and the data were normalized to those of glyceraldehyde 3‐phosphate dehydrogenase. The specific primer sequences were listed in Table S1.

### 2.11 Methylation‐Specific PCR (MSP) Assays

MSP assays assessed the DNA methylation status. Genomic DNA was extracted from the placentas and HTR‐8/SVneo cells using DNA extraction kits (TIANGEN BioTech,Beijing,Chian) according to the manufacturer's instructions. Bisulfite conversion of DNA was performed using EZ DNA methylation Gold (ZYMO Research Corp., Irvine, CA, USA). Methylation‐specific primers were designed to selectively amplify methylated or unmethylated DNA sequences in the *TP53BP2* promoter region. After amplification, PCR products were analyzed via 2% gel electrophoresis to visualize the presence or absence of specific bands corresponding to methylated or unmethylated DNA, respectively. The primer sequences used for the MSP assays were listed in Table S2.

### 2.12 Bisulfite‐Sequencing PCR (BSP)

DNA from the MSP results was subjected to sodium bisulfite treatment using a DNA bisulfite kit (Qiagen, Beijing, China) according to the manufacturer's instructions. After bisulfite conversion, DNA was purified and recovered using a SanPrep Column DNA Gel Extraction Kit (Sangon Biotech, Shanghai, China). The primers for bisulfite pyrosequencing were designed to target the specific region of interest and were used with 3730 sequencing analyzers. The primers for the *TP53BP2* methylation reaction were as follows: *TP53BP2* forward 5´‐AACTTCACGGTGGGTTTCAAGC‐3´ and *TP53BP2* reverse 5´‐GTGCAGGCCTGAGCCTTCTG GC‐3´.

### 2.13 Co‐Immunoprecipitation (Co‐IP)

Cells were washed thrice with PBS and subsequently lysed on ice in lysis buffer (Beyotime Biotechnology, Shanghai, China). Next, the lysates were centrifuged at 12,000 ×*g* for 15 min. Subsequently, cell lysates were incubated with specific antibodies for 1 h, followed by a 30‐min incubation with Dynabeads Protein G beads (Thermo Fisher Scientific) at 4°C. The beads were washed thrice with cold lysis buffer. After the washes, the beads were boiled in loading buffer (10 µL, 5×; Beyotime Biotechnology) for 5 min before being analyzed by western blotting using antibodies against *DNMT1*, *G9a*, *E2F1*, Flag, Myc, and glutathione *S*‐transferase (GST).

### 2.14 Chromatin Immunoprecipitation (ChIP) Assay

ChIP assays were performed according to the manufacturer's instructions (Millipore, Massachusetts, USA). Antibodies against *H3K4me1*, *H3K4me2*, *H3K4me3*, *H3K9me2*, *H3K9me3*, *H3K36me3*, *H3K27me3*, *G9a*, *DNMT1*, or *E2F1* were used for ChIP. RT‐PCR was performed using specifically designed qPCR primers targeting the proximal promoter region of *TP53BP2*. IgG was used as a negative control to measure nonspecific background signals through immunoprecipitation. The resulting amplified product was evaluated by electrophoresis, and the signals were quantified as a percentage of the input. The primer sequences were listed in Table S3.

### 2.15 Luciferase Reporter Assay

Luciferase reporter assay was performed to determine the activity of the *TP53BP2* promoter. For the promoter activity assay, various fragments of the TP53BP2 promoter (−35/+1, −599/+1, −1018/+1, −1530/+1, −599/+641, and −2000/+1) were inserted into the pGL3‐Basic plasmid. Cells were seeded in 24‐well plates and co‐transfected with the aforementioned reporter constructs and a *Renilla* luciferase reporter plasmid. After a 48‐h transfection period, luciferase activities were quantified using a dual‐luciferase reporter assay system (Promega, Madison, WI, USA). Firefly luciferase activity was determined and normalized to *Renilla* luciferase activity. The data reported represent the average of three independent experiments.

### 2.16 Statistical Analysis

Data from three replicates per experiment were summarized as the mean ± standard deviation. Statistical analysis involved a one‐way analysis of variance, followed by the Student's–Newman–Keuls’ test for multiple comparisons within treatment groups or Student's *t‐*test for comparisons between two groups. Receiver operating characteristic (ROC) curves and the corresponding area under the curve (AUC) were generated using the R package “pROC” to assess the diagnostic value of *TP53BP2*. A significance threshold of *P* <0.05 was applied.

## 3 Results

### 3.1 Identification and Validation of TP53BP2 in Placental Trophoblasts in PE

**FIGURE 1:**
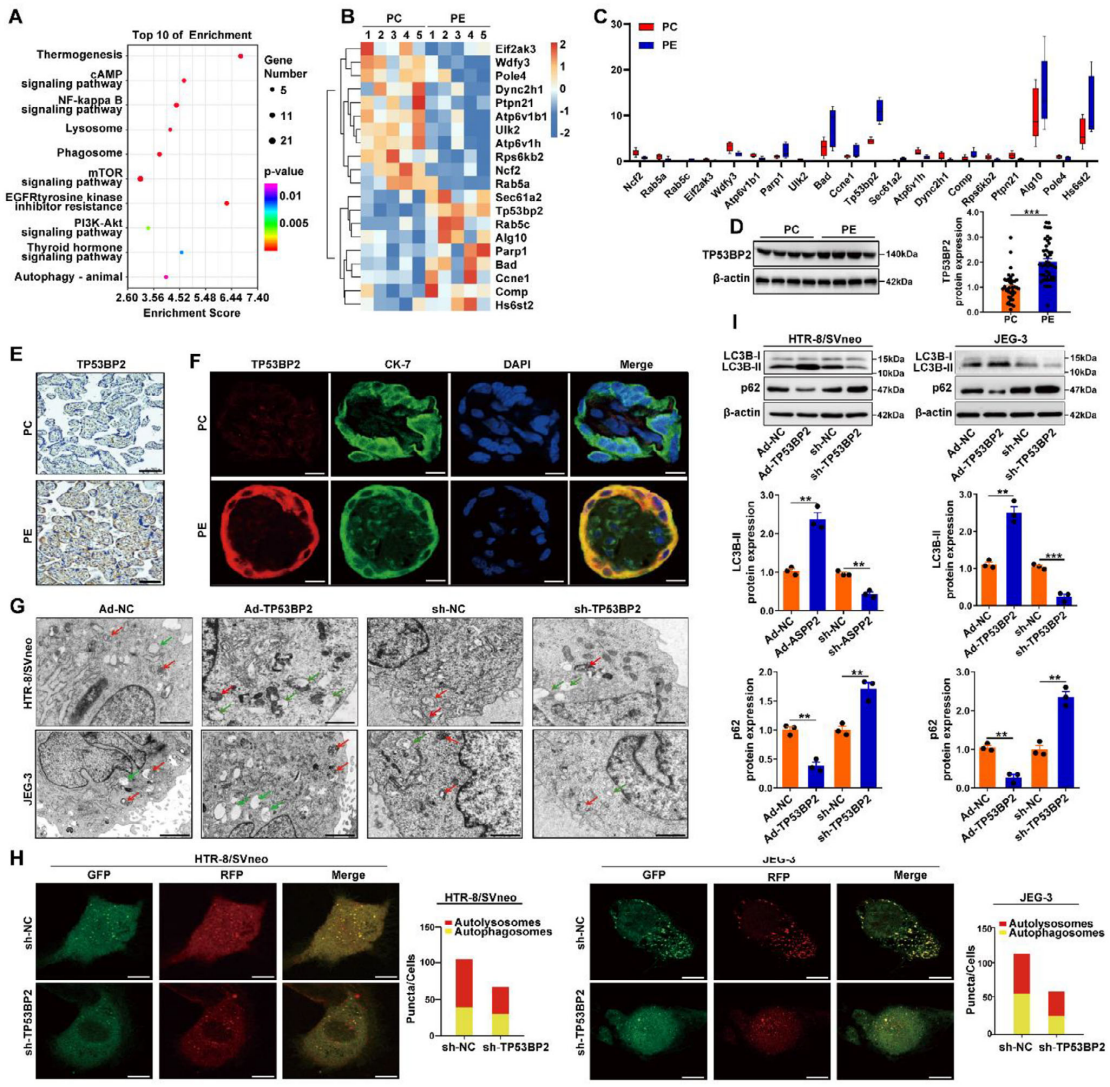
TP53BP2 was upregulated in placental trophoblasts from PE pregnancies. **(A)** KEGG signaling pathway histogram. **(B)** Cluster heatmap showing the top 20 autophagy‐related DEGs associated with autophagy (|fold change|≥2.0, *P*≤0.05) in the placentas of PE and non‐PE pregnancies. Red and blue strips indicate upregulated and downregulated genes, respectively. **(C)** Box plot illustrating the expression of the top 20 DEGs in placentas from PE and non‐PE pregnancies. The blue and red box plots represent PE and non‐PE pregnancies, respectively. **(D)** The expression of *TP53BP2* in placentas was detected via western blotting (PC, n = 40; PE, n = 45). **(E)** Representative immunohistochemical staining of *TP53BP2*. Scale bar = 200 µm. **(F)** Double immunofluorescence staining showing colocalization of *TP53BP2* (red) and CK‐7 (trophoblast marker, green). The nuclei were stained with DAPI (blue). Scale bar = 50 µm. **(G)** Autophagosomes in HTR8/Svneo and JEG‐3 cells transfected with Ad‐*TP53BP*2 or sh‐*TP53BP2* under hypoxic conditions were observed using transmission electron microscopy (TEM). Scale bar = 1000 nm. **(H)** Representative images of GFP‐RFP‐LC3 staining in HTR8/Svneo and JEG‐3 cells transfected with sh‐*TP53BP2* under hypoxic conditions (n = 3). Scale bar = 20 µm. **(I)** The expression of *LC3B‐II* and *p62* in HTR8/Svneo and JEG‐3 cells transfected with Ad‐*TP53BP2* or sh‐*TP53BP2* under hypoxic conditions was detected by western blotting (n = 3). Data are presented as mean ± SD. Student's *t*‐test (unpaired, two‐tailed) was used to compare two independent groups. ^**^ *P*<0.01, ^***^ *P*<0.001.

To elucidate the molecular mechanisms underlying autophagy in placental trophoblasts in early onset PE, RNA‐seq was used to examine autophagy‐related differentially expressed genes (DEGs) in placentas derived from early onset PE pregnancies compared to those from non‐PE pregnancies. Functionally related genes were predominantly enriched in autophagy‐related signaling pathways, such as the phosphatidylinositol 3‐kinase/Akt, AMPK, and mammalian target of rapamycin (mTOR) signaling pathways (Figure 1A). Cluster heatmaps showed the top 20 DEGs involved in autophagy‐related signaling pathways in placentas from PE pregnancies (Figure 1B), and *TP53BP2* was the most robust inducer among them (Figure 1C). Moreover, *TP53BP2* expression was markedly higher in the placentas of PE pregnancies than in those of non‐PE pregnancies (Figure 1D). IHC and immunofluorescence staining further revealed *TP53BP2* upregulation in trophoblasts from PE pregnancies (Figure 1E,F). To investigate the functional role of *TP53BP2*, we conducted *TP53BP2* overexpression and knockdown in HTR8/Svneo and JEG‐3 cells by transfecting Ad‐ *TP53BP2* or three independents sh‐ *TP53BP2* constructs, respectively. The results showed that *TP53BP2* mRNA and protein levels were significantly increased following Ad‐*TP53BP2* transfection. Among the three independent sh‐*TP53BP2* constructs, sh‐ *TP53BP2* (#3) exhibited the highest knockdown efficiency in both HTR8/Svneo and JEG‐3 cells compared to sh‐ *TP53BP2* (#1 and #2) (Figure S1). Therefore, sh‐ *TP53BP2* (#3) was used in all subsequent experiments in this study. The TEM results revealed increased autophagosome and autolysosome formation in trophoblasts transfected with Ad‐*TP53BP2*, and the opposite effect was observed in trophoblasts transfected with sh‐*TP53BP2* (Figure 1G). An autophagic flux assay using tandem fluorescent GFP‐RFP‐LC3 revealed decreased total autophagosome and autolysosome formation in trophoblasts transfected with sh‐*TP53BP2* (Figure 1H). *LC3B‐II* and *p62* are complementary autophagy markers, with *LC3B‐II* reflecting autophagosome formation and *p62* indicating the process of substrate recognition and degradation. Therefore, *LC3B‐II* and *p62* protein expression levels were assayed to determine the extent of autophagy. Western blotting analysis confirmed the effects of *TP53BP2* on *LC3B‐II* and *p62* levels in HTR8/SVneo and JEG‐3 cells under hypoxia (Figure 1I). These results demonstrate that *TP53BP2* upregulation increases trophoblast autophagy in PE placentas.

### 3.2 TP53BP2 Silencing Attenuates PE Progression by Reducing Autophagy in Rats

**FIGURE 2:**
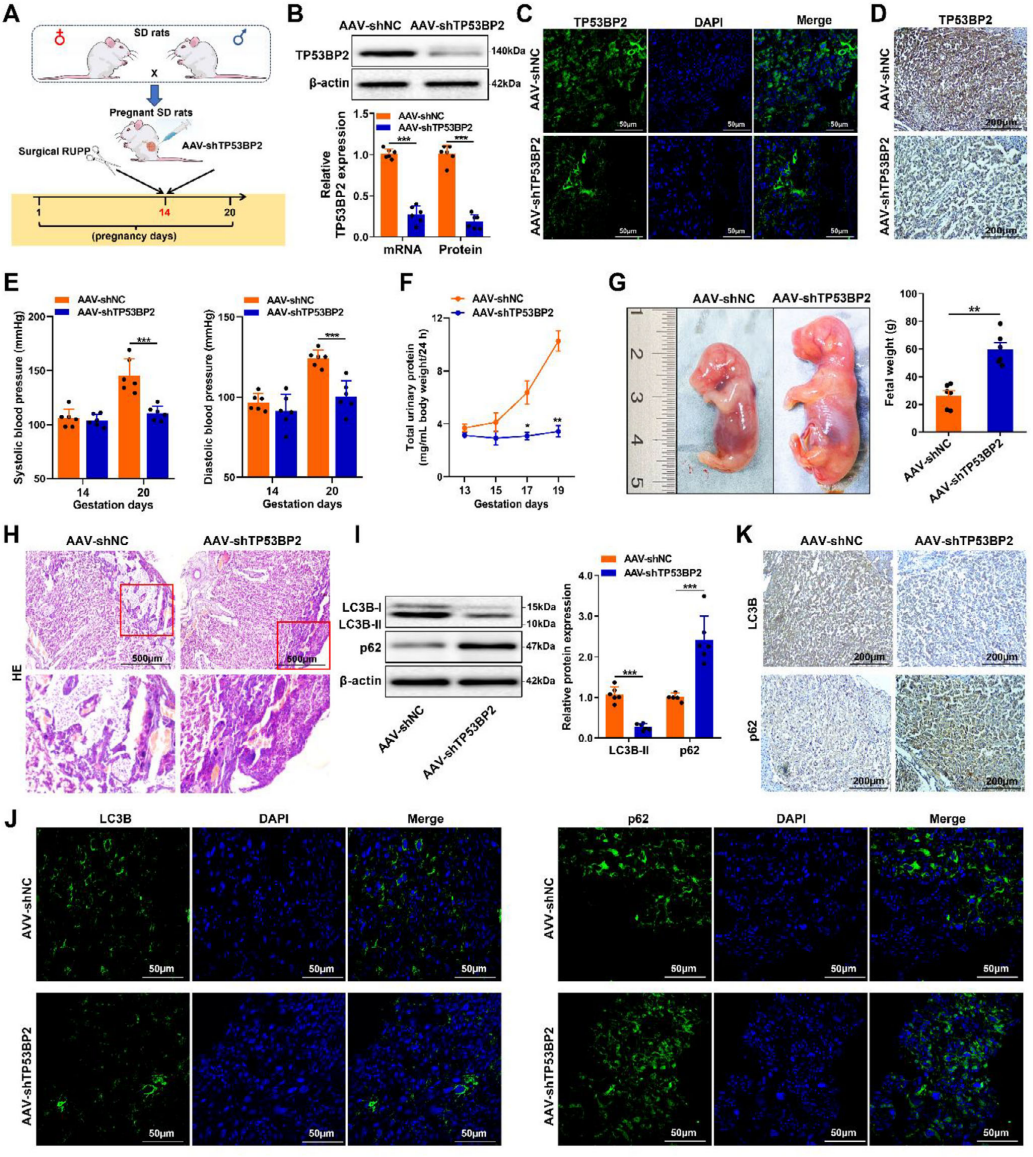
TP53BP2 is a potential therapeutic target for trophoblast autophagy in PE. **(A)** On gestational day 14, SD rats underwent surgical reduction of uterine perfusion pressure (RUPP), followed by a single injection of 10 µL of AAV‐*shTP53BP2* (1.5E+11) into the placenta. **(B)** Expression of *TP53BP2* in the placentas of preeclamptic rats was detected by western blotting and qRT‒PCR. **(C)** Immunofluorescence staining showing *TP53BP2* expression in the placentas of preeclamptic rats. Scale bar = 50 µm. **(D)** Immunohistochemical staining was used to detect the expression of *TP53BP2* in the placentas of preeclamptic rats. Scale bar = 200 µm. **(E)** Systolic and diastolic blood pressure were measured via a noninvasive tail‐cuff blood pressure measurement system in preeclamptic rats. **(F)** Total urine protein levels were measured using a protein assay in preeclamptic rats. **(G)** Gross appearance and birth weight of the fetus at embryonic day 18.5 (E18.5). **(H)** H&E staining analysis of placental pathological changes in preeclamptic rats. Scale bar = 500 µm. **(I)** The expression of *LC3B*‐II and *p62* in the placentas of preeclamptic rats was detected by western blotting. **(J)** Immunofluorescence staining was used to detect the expression of *LC3B* and *p62* in the placentas of preeclamptic rats. Scale bar = 50 µm. **(K)** Immunohistochemical staining was used to detect the expression of LC3B and p62 in the placentas of preeclamptic rats. Scale bar = 200 µm. Data are presented as mean ± SD. Student's *t*‐test (unpaired, two‐tailed) was used to compare two independent groups. ^*^ *P*<0.05, ^**^ *P*<0.01, ^***^ *P*<0.001.

Based on these in vitro findings, a PE model was established by inducing RUPP in rats on GD14 to investigate the role of *TP53BP2* (Figure 2A–D). *TP53BP2* knockdown via the use of recombinant AAV serotype 9 vectors carrying *TP53BP2* shRNA (AAV‐*shTP53BP2*) in PE rats resulted in decreased BP (Figure 2E) and proteinuria (Figure 2F) and increased fetal weight (Figure 2G). Hematoxylin and eosin (H&E) staining also revealed a reduction in the hydropic degeneration of decidual cells and deposition of fibrous proteins in this rat model (Figure 2H). Furthermore, placentas from PE rats with downregulated *TP53BP2* exhibited reduced *LC3B*‐II expression and increased *p62* expression (Figure 2I). Similar results were obtained by immunofluorescence staining (Figure 2J). In addition, IHC staining revealed a significant reduction in *LC3B* expression and upregulation of *p62* expression in the placentas of PE rats subjected to *TP53BP2* knockdown (Figure 2K). Collectively, these results suggest that *TP53BP2* knockdown attenuates autophagy in trophoblasts in the placentas of PE rats.

### 3.3 TP53BP2 Enhances Trophoblast Autophagy by Regulating Beclin‐1 Expression

**FIGURE 3:**
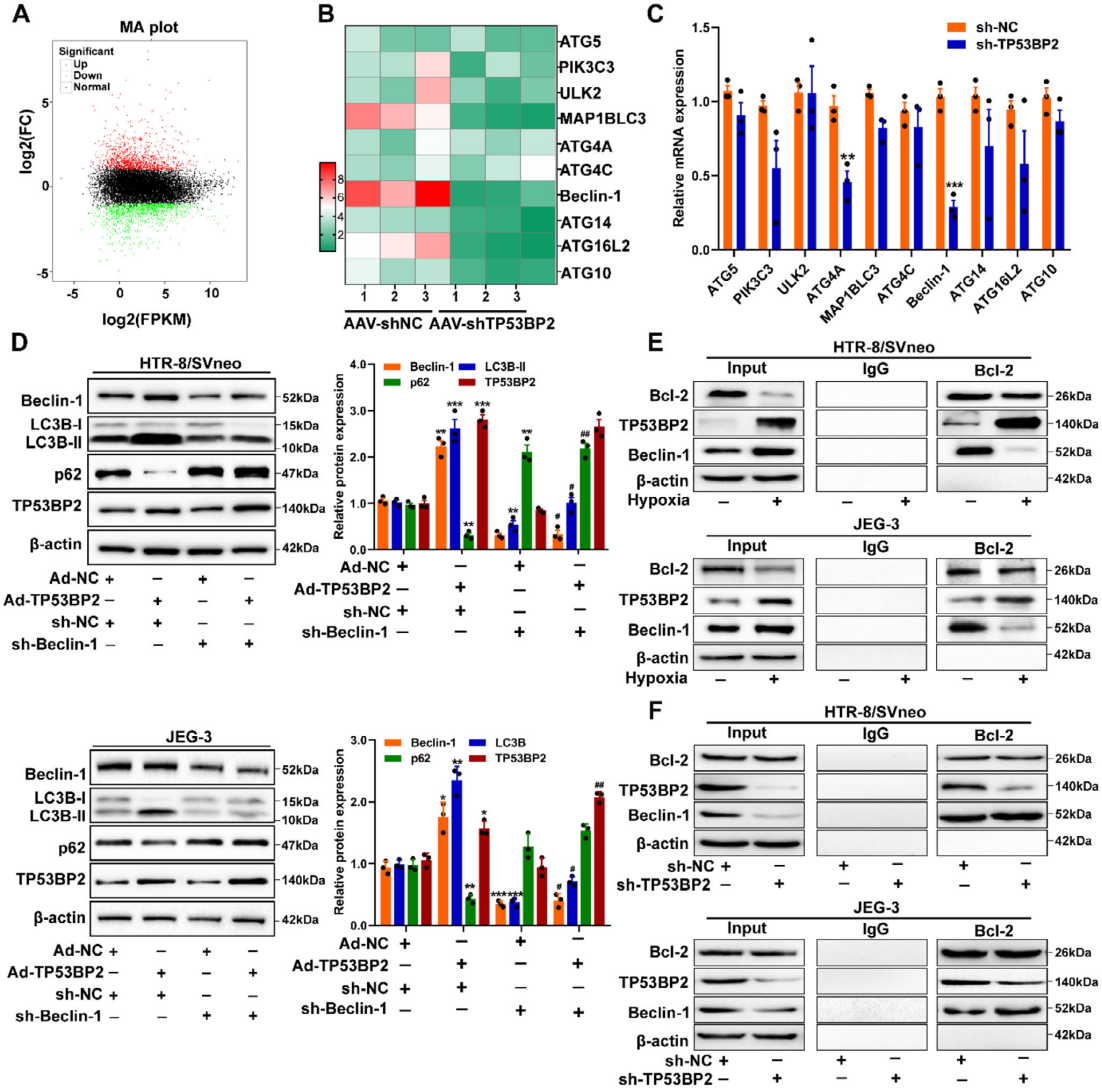
TP53BP2 enhances trophoblast autophagy by regulating Beclin‐1 expression. **(A)** Volcano plot of 2081 DEGs in HTR8/SVneo cells transfected with sh‐TP53BP2. The red dots and green dots indicate upregulated and downregulated gene expression, respectively (|fold change|≥2.0, *P*≤0.05), and the black dots indicate unchanged genes. **(B)** Heatmap of the top 10 downregulated autophagy‐related genes in the placentas of preeclamptic rats injected with AAV‐*shTP53BP2*. **(C)** qRT‒PCR validation of the top 10 downregulated genes. **(D)** Expression of *Beclin*‐1, *LC3B*‐II, *p62* and *TP53BP2* in HTR8/SVneo and JEG‐3 cells transfected with Ad‐TP53BP2 and/or sh‐Beclin‐1 under hypoxic conditions was detected by western blotting. **(E)** Co‐immunoprecipitation (Co‐IP) assay followed by immunoblotting showing the interactions of *Bcl*‐2 with *TP53BP2* and *Beclin*‐1 in HTR8/SVneo and JEG‐3 cells under hypoxic conditions.Cell lysates were subjected to immunoprecipitation with an anti‐*Bcl*‐2 antibody. **(F)** Co‐IP assay followed by immunoblotting showing the interactions of *Bcl*‐2 with *TP53BP2* and Beclin‐1 in HTR8/SVneo and JEG‐3 cells transfected with sh‐*TP53BP2* under hypoxic conditions. Data are presented as mean ± SD. Student's *t*‐test (unpaired, two‐tailed) was used to compare two independent groups, and a two‐way ANOVA test was performed for comparisons of multiple groups. ^*^ *P*<0.05, ^**^ *P*<0.01, ^***^ *P*<0.001; ^#^ *P*<0.05, ^##^ *P*<0.01.

Next, RNA‐seq on HTR8/SVneo cells with *TP53BP2* knockdown was performed to investigate the molecules involved in autophagy. The results revealed the upregulation of 1046 molecules and downregulation of 1035 molecules (|fold change|≥2.0; *P*≤0.05; Figure 3A). Gene Ontology analysis revealed that these molecules are involved in autophagy, ATP binding, protein serine/threonine kinase activity, mTOR signaling, HIF1 signaling pathway, and AMPK signaling (Figure S2). A qRT‐PCR assay was subsequently performed to measure the levels of the top 10 downregulated genes. Results revealed a significant reduction in the level of *Beclin*‐1, a key marker of autophagy, in the placentas of PE rats (Figure 3B,C). To further confirm the role of *Beclin*‐1 in *TP53BP*‐induced autophagy, three independent sh‐*Beclin*‐1 was constructs were created and transduced into HTR8/SVneo and JEG‐3 cells. The results revealed that the *Beclin*‐1 mRNA and protein expression levels were significantly decreased, following transduction with sh‐ *Beclin*‐1 (#1) (Figure S3). In Figure 3D, co‐transfection with sh‐*Beclin*‐1 and Ad‐*TP53BP2* resulted in decrease in LC3B‐II levels and increased in *p62* expression in HTR8/SVneo and JEG‐3 cells. *Bcl*‐2 interacts with *Beclin*‐1, an autophagy initiator. Previous studies have indicated that the release of *Beclin*‐1 from the *Bcl*‐2‐*Beclin*‐1 complex initiates *TP53BP2*‐induced autophagy. The present study investigated the role of *Bcl*‐2 binding to the BH3 domain of *Beclin*‐1 in *TP53BP2*‐induced autophagy in trophoblasts. CoIP revealed an increase in the interaction between *TP53BP2* and *Bcl*‐2, whereas a disruption of the interaction between *Beclin*‐1 and *Bcl*‐2 was observed in HTR8/SVneo and JEG‐3 cells under hypoxic conditions (Figure 3E). Conversely, *TP53BP2* knockdown increased the interaction between *Beclin*‐1 and *Bcl*‐2 (Figure 3F). These results demonstrate that *TP53BP2* knockdown inhibits trophoblast autophagy by decreasing Beclin‐1 expression and promoting the interaction between *Beclin*‐1 and *Bcl*‐2.

### 3.4 TP53BP2 is Associated With the Clinicopathological Characteristics of PE

**FIGURE 4:**
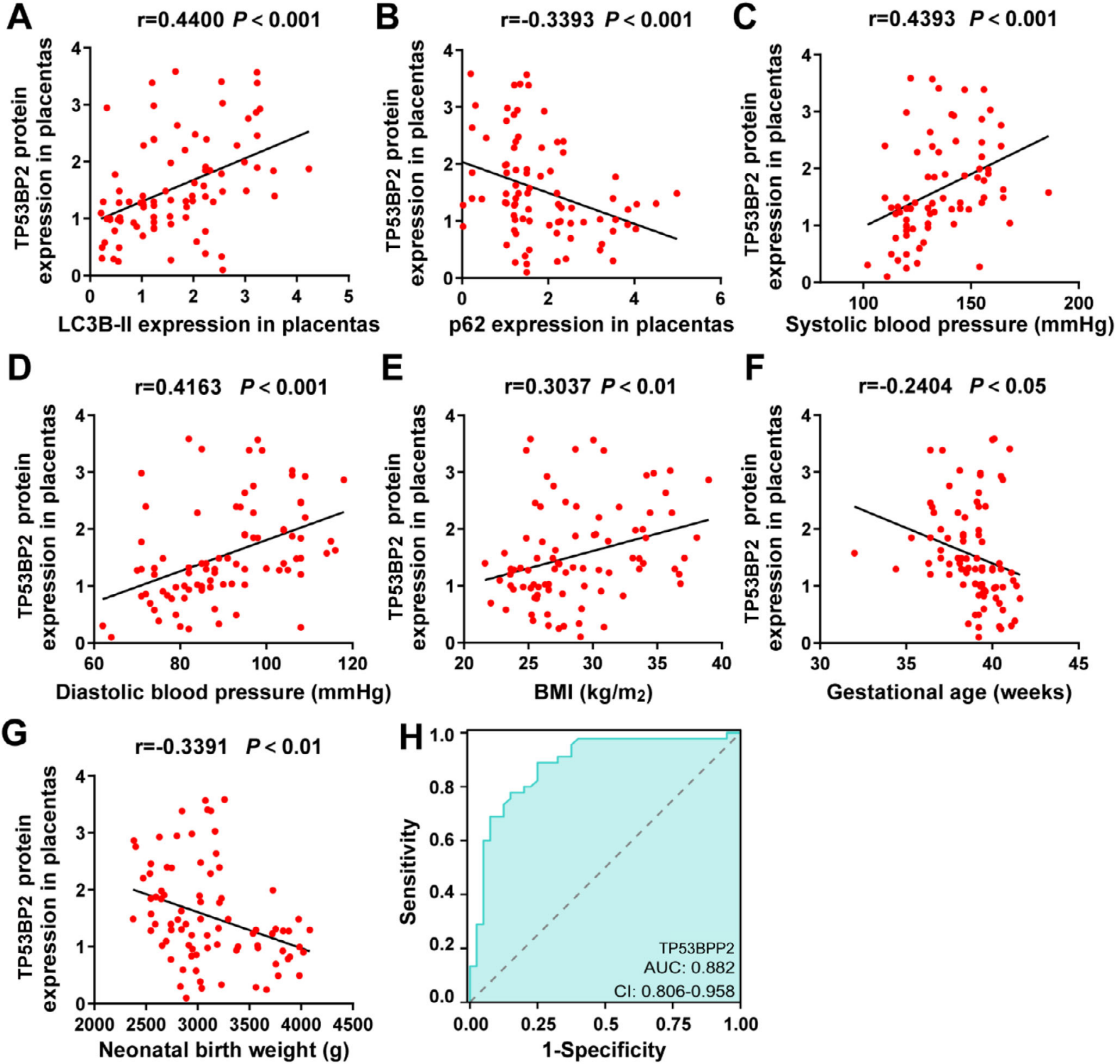
Correlation between TP53BP2 expression and clinicopathological characteristics of patients with PE. **(A, B)** Pearson's correlation analysis between *TP53BP2* expression and *LC3B*‐II or *p62* levels in placentas from PE and non‐PE pregnancies. **(C–G)** Pearson's correlation analysis between *TP53BP2* expression and systolic blood pressure **(C)**, diastolic blood pressure **(D)**, BMI **(E)**, gestational age **(F)**, and neonatal birth weight **(G)** in PE pregnancies and non‐PE pregnancies. **(H)** The cutoff value, sensitivity, and specificity were established using receiver operating characteristic (ROC) curves to evaluate the diagnostic value of *TP53BP2* in PE pregnancies. The data are presented as the means ± SDs. ^**^ *P*<0.01.

To explore the clinical significance of *TP53BP2* in PE progression, 85 placentas were collected from early onset PE pregnancies (<34 weeks of gestation; n = 45) and non‐PE pregnancies (n = 40). The pathological characteristics of these pregnancies are presented in Table 1. There was no significant difference in maternal age or fetal sex between the PE and non‐PE pregnancies (*P* >0.05). However, SBP, DBP, and proteinuria were significantly increased in PE pregnancies (*P<*0.0001). Additionally, there was a significant difference in the maternal body mass index (BMI) between the PE and non‐PE pregnancies (*P*< 0.05). Moreover, compared to non‐PE pregnancies, PE pregnancies were associated with decreased gestational age at delivery (GAD) and lower neonatal birth weight (NBW; *P*<0.05), indicating that these clinicopathological parameters were in accordance with the diagnostic criteria. To evaluate the significance of *TP53BP2* in placental dysfunction, the correlation among *TP53BP2* and *LC3B‐II* and *p62* was analyzed in the placentas. The results revealed a positive association between *TP53BP2* and *LC3B‐II* levels in PE and non‐PE pregnancies and a negative correlation with *p62* (Figure 4A,B). Additionally, *TP53BP2* levels in placentas were positively correlated with SBP, DBP, and BMI but negatively correlated with GAD and NBW (Figure 4C–G). Furthermore, ROC analysis demonstrated that *TP53BP2* had the highest AUC value of 0.882 for diagnosing PE pregnancies (Figure 4H). Therefore, these results suggest that *TP53BP2* may be a predictive biomarker associated with the clinicopathological characteristics of pregnancies complicated by PE.

**TABLE 1:** Clinical data of non‐PE pregnancies and early‐onset PE pregnancies.

Characteristic	PC (n=40)	PE (n=45)	*P*‐value
Maternal age (years)	28.13±4.28	27.49±4.63	*P=*0.4818
Gestational age (weeks)	39.57±1.11	38.43±2.05	*P=*0.0022
BMI (kg/m^2^)	28.62±0.57	30.44±0.63	*P=*0.0374
Systolic blood pressure (mmHg)	113.0±1.53	145.0±2.18	*P*<0.0001
Diastolic blood pressure (mmHg)	73.0±1.20	97.0±1.30	*P*<0.0001
Urine protein/24 h (g)	N/A	2.86±0.31	N/A
Neonatal birth weight (g)	3384±65.10	3049±106.80	*P=*0.0111
Fetal gender (male/female)	17/23	26/19	*P=*0.3316

BMI: body mass index.

### 3.5 DNMT1‐Mediated DNA Methylation Inhibits TP53BP2 Transcription

**FIGURE 5:**
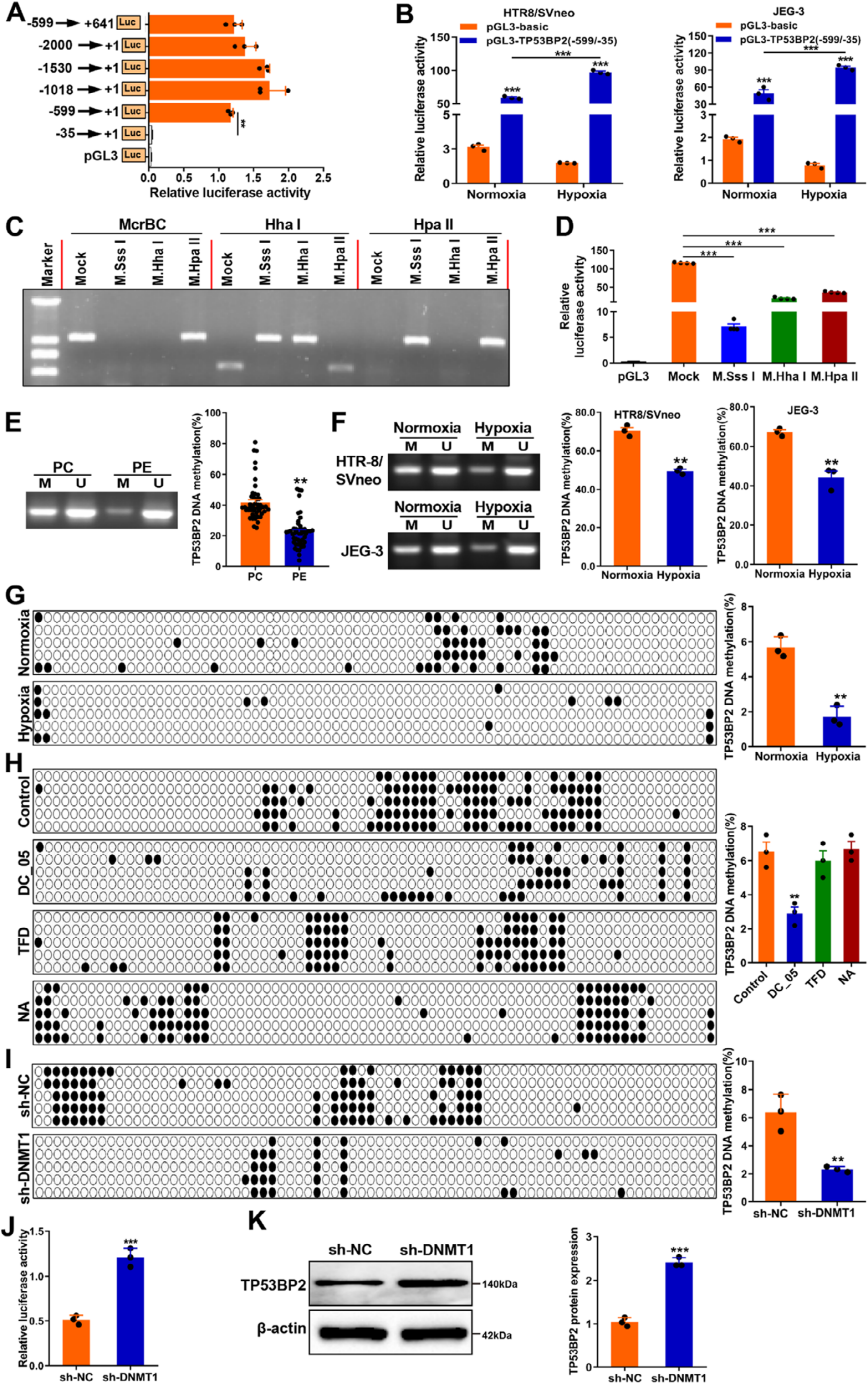
DNA methylation represses TP53BP2 transcription via DNMT1. **(A)** The promoter activity of *TP53BP2* was evaluated using a dual‐luciferase reporter assay. Different fragments of the *TP53BP2* promoter (‐35/+1, ‐599/+1, ‐1018/+1, ‐1530/+1, ‐599/+641, and ‐2000/+1) were transfected into HEK293T cells with a Renilla luciferase vector (internal control), and the results are presented as firefly luciferase activity normalized to Renilla luciferase activity. **(B)** Dual‐luciferase reporter assay analysis of the luciferase activities of the *TP53BP2* promoter (‐599/‐35) in HTR8/SVneo and JEG‐3 cells subjected to hypoxic conditions. **(C)** Methylation of the *TP53BP2* promoter in HEK293T cells. Following methylation with *SssI*, *HhaI*, or *HpaII* methylases, *TP53BP2* promoter fragments were digested with McrBC (a methylation‐specific restriction enzyme), *HpaII* or *HhaI* (a methylation‐sensitive restriction enzyme) to confirm the methylation status of the TP53BP2 promoter construct. **(D)** The activity of the *TP53BP2* proximal promoter methylated with *SssI*, *HhaI*, or *HpaII* methylases was assessed using a luciferase reporter assay in HEK293T cells transfected with luciferase reporter constructs. **(E, F)** The DNA methylation levels of the *TP53BP2* promoter were evaluated using methylation‐specific PCR (MSP) in placentas and in HTR8/SVneo and JEG‐3 cells under hypoxic conditions. U: unmethylated; M: methylated. **(G)** The DNA methylation levels of the *TP53BP2* promoter were evaluated via bisulfite sequencing PCR (BSP) in HTR8/SVneo cells under hypoxic conditions. White cycle, unmethylated CpG dinucleotides; black cycle, methylated CpG dinucleotides. The percentage of methylation at each CpG dinucleotide was calculated as the number of methylated clones at each CpG site divided by the total number of clones at the same CpG site and is shown in the right panel. **(H)** The DNA methylation level of the *TP53BP2* promoter was evaluated via BSP in HTR8/SVneo cells treated with DC_05 (a *DNMT1*‐specific inhibitor), Theaflavin‐3, 3’‐digallate (TFD, a *DNMT3a*‐specific inhibitor) or Nanomycin A (NA, a *DNMT3b*‐specific inhibitor) under hypoxic conditions. **(I)** The DNA methylation level of the *TP53BP2* promoter was evaluated via BSP in HTR8/SVneo cells transfected with sh‐*DNMT1* under hypoxic conditions. **(J)** Transcriptional activity of *TP53BP2* in HTR8/SVneo cells transfected with sh‐*DNMT1*. **(K)** *TP53BP2* expression was evaluated by western blotting in HTR8/SVneo cells transfected with sh‐*DNMT1* under hypoxic conditions. Data are presented as mean ± SD. Student's *t*‐test (unpaired, two‐tailed) was used to compare two independent groups. ^*^ *P*<0.05, ^**^ *P*<0.01, ^***^ *P*<0.001.

First, the genomic sequence of the *TP53BP2* promoter was analyzed to evaluate the effects of epigenetic regulation on *TP53BP2* expression using the University of California at Santa Cruz (UCSC) database. As expected, the *TP53BP2* promoter contained a high percentage of GC bases in its CpG islands (Figure S4A). The MethPrimer program identified a single CpG island measuring 1240‐bp long. This island spans positions −599 to +641 relative to the transcription start site. It has a CG content of 50% and a CpG ratio of 0.6. This CpG island is located at the distal end of the 5´‐flanking region of *TP53BP2* (Figure S4B), which may regulate *TP53BP2* levels through methylation. Several fragments of the *TP53BP2* 5‐flanking region were subsequently inserted into the firefly luciferase vector pGL3, and luciferase activity assay results revealed that the −599/−35 fragment, which spans most CpG dinucleotides of the *TP53BP2* promoter, exhibited the highest promoter activity (Figure 5A). Consistent with this result, a luciferase assay revealed increased transcription activity of *TP53BP2* in HTR8/SVneo and JEG‐3 cells after transfection with a luciferase reporter of pGL3 harboring the fragment (−599/−35; Figure 5B), indicating that this region (−599/−35) is the core regulatory region for *TP53BP2*. To examine whether DNA methylation directly represses *TP53BP2* promoter activity, the *TP53BP2* proximal promoter region was cloned from −599/−35. The cloned inserts were methylated using the methylases *SssI* (M.SssI), HhaI (M.HhaI), and *HpaII* (M.HhaII). *SssI* was adopted to methylate all 51 CpG sites within the sequence 5´‐CpG‐3´, *HhaI* methylated only nine CpG sites within the sequence 5´‐GCGC‐3´, and *HpaII* methylated three CpG sites within the sequence 5´‐CCGG‐3´. The proper methylation of the fragments was confirmed by digestion with the restriction enzymes McrBC (methylation‐specific restriction enzyme), *HhaI*, and *HpaII* (methylation‐sensitive restriction enzyme; Figure 5C). Transfection of trophoblasts with a luciferase reporter vector and subsequent luciferase assay revealed that treatment with the three methylases reduced the *TP53BP2* promoter activity. Notably, SssI methylase had the most significant inhibitory effect (Figure 5D). Next, differences in *TP53BP2* DNA methylation levels were detected using MSP. As shown in Figure 5E,F, global DNA methylation levels were decreased in the placentas of PE pregnancies and trophoblasts under hypoxic conditions. BSP further revealed a remarkable decrease in DNA methylation levels within the −599/−35 region of the *TP53BP2* promoter in HTR8/SVneo cells exposed to hypoxic conditions (Figure 5G). These results revealed that DNA hypomethylation modulates the transcriptional activation of *TP53BP2* in trophoblasts in the placentas of PE pregnancies.

To determine the key enzymes involved in DNA methylation, HTR8/SVneo cells were treated with DC_05 (*DNMT1* inhibitor), theaflavin‐3,3´‐digallate (TFD; *DNMT3a* inhibitor), or nanomycin A (NA; *DNMT3b* inhibitor) under hypoxic conditions. In Figure 5H, the DC_05, but not TFD or NA, treatment caused a significant decrease in the DNA methylation level of the *TP53BP2* promoter in HTR8/SVneo cells under hypoxic conditions. To further confirm the role of *DNMT1* in regulating *TP53BP2* DNA methylation, we generated HTR8/SVneo cells with *DNMT1* knockdown by transducing them with three independent sh‐ *DNMT1* constructs. The results showed that *DNMT1* mRNA and protein expression were significantly reduced following transduction with sh‐ *DNMT1* (#1) (Figure S5). The results revealed that *DNMT1* knockdown decreased the DNA methylation level of the *TP53BP2* promoter (Figure 5I) and increased the transcriptional activity and protein levels of *TP53BP2* (Figure 5J,K). These results suggest that *DNMT1*‐mediated DNA methylation strongly inhibits *TP53BP2* expression.

### 3.6 DNMT1 Inhibited TP53BP2 Expression by Inversely Modulating E2F1

**FIGURE 6:**
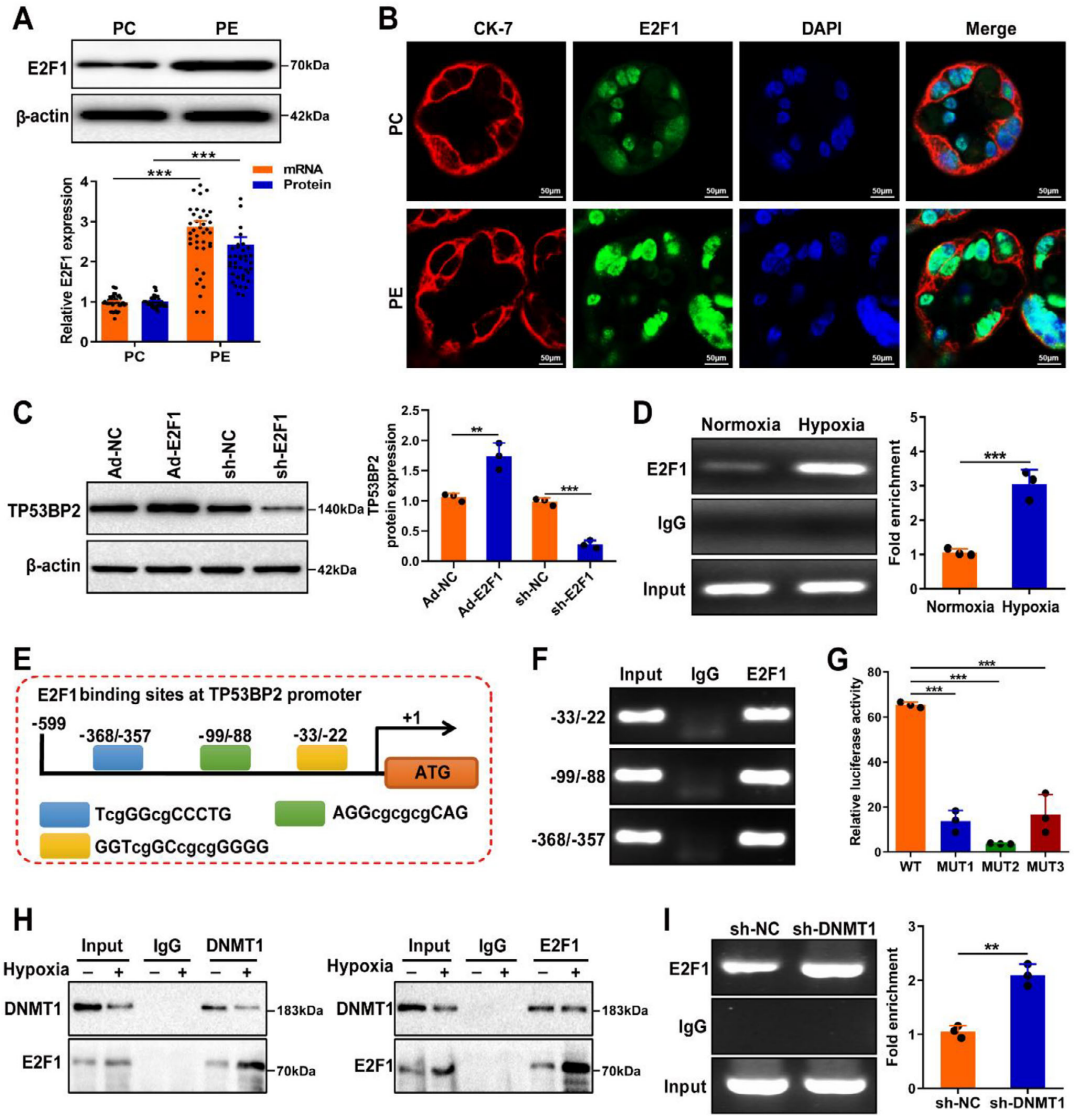
DNMT1 inhibits TP53BP2 expression by inversely modulating E2F1 in trophoblasts. **(A)** *E2F1* expression in the placenta was detected using qRT‒PCR and western blotting. **(B)** Immunofluorescence staining was used to detect *E2F1* expression (green) in placental trophoblasts. Scale bar = 50 µm. **(C)** *TP53BP2* expression was detected by western blotting in HTR8/SVneo cells transfected with Ad‐*E2F1* or sh‐*E2F1* under hypoxic conditions. **(D)** Enrichment of *E2F1* at the promoter region of TP53BP2 in HTR8/SVneo cells under hypoxic conditions was examined using a ChIP assay with an *E2F1* antibody. **(E)** A schematic diagram of the predicted *E2F1*‐binding sites in the *TP53BP2* promoter region from the JASPAR database (http://www.genereg.net/). The blue box represents the ‐33/‐22 site. The green box represents the ‐99/‐88 site. The yellow box represents the ‐368/‐357 site. **(F)** A ChIP assay was conducted to assess the binding of *E2F1* to specific sites (‐33/‐22, ‐99/‐88, and ‐368/‐357) on the *TP53BP2* promoter in HTR8/SVneo cells subjected to hypoxic conditions. **(G)** The promoter activities of *TP53BP2* with the wild‐type (WT) or mutant ‐33/‐22 site (Mut1), ‐99/‐88 site (Mut2) and ‐368/‐357 site (Mut3) of *E2F1* were determined via a luciferase reporter assay in HEK293T cells. **(H)** Co‐IP assay followed by immunoblotting showing the interactions between *DNMT1* and *E2F1* in HTR8/SVneo cells under hypoxic conditions. **(I)** A ChIP assay was performed to demonstrate that *E2F1* binds to the *TP53BP2* promoter in HTR8/SVneo cells transfected with sh‐*DNMT1*. Data are presented as mean ± SD. Student's *t*‐test (unpaired, two‐tailed) was used to compare two independent groups. ^**^ *P*<0.01, ^***^ *P*<0.001.

*TP53BP2* has been reported to be a direct target of *E2F1*. In this study, qRT‐PCR and western blotting assays revealed a significant increase in *E2F1* levels in the placentas of PE pregnancies compared to those of non‐PE pregnancies (Figure 6A). This finding was further confirmed by the immunofluorescence staining of trophoblasts from the placentas of PE pregnancies (Figure 6B). Next, we examined the impact of E2F1 on TP53BP2 expression by constructing *E2F1* overexpression or knockdown in HTR8/Svneo cells. The results showed that *E2F1* mRNA and protein levels were significantly increased following Ad‐ *E2F1* transfection. Among the three independent sh‐*E2F1* constructs, sh‐*E2F1* (#3) exhibited the highest knockdown efficiency in both HTR8/Svneo cells compared to sh‐ *E2F1* (#1 and #2) (Figure S6). *E2F1* overexpression and knockdown increased and inhibited *TP53BP2* expression, respectively, in HTR8/SVneo cells subjected to hypoxic conditions (Figure 6C). Furthermore, a ChIP assay using an anti‐*E2F1* antibody revealed significant enrichment of *E2F1* at the *TP53BP2* promoter under hypoxic conditions (Figure 6D). Next, we used the JASPAR database to compute putative transcription factor‐binding elements encompassing the hypomethylated CpG site in the *TP53BP2* promoter. Three putative *E2F1* binding sites (−33/−22, −99/−88, and −368/−357) were identified in the *TP53BP2* promoter (Figure 6E). A ChIP assay revealed remarkable binding of *E2F1* to the *TP53BP2* promoter at the 33/22, 99/88, and 368/357 sites (Figure 6F). Additionally, a luciferase reporter assay revealed a significant decrease in the activity of the *TP53BP2* promoter after mutation of the 33/22 (*Mut1*), 99/88 (*Mut2*), and 368/357 (*Mut3*) sites (Figure 6G). Methyltransferases can regulate target gene expression by directly interacting with transcription factors [33], and the CoIP method was adopted to investigate the relationship between *DNMT1* and *E2F1*. Consistent with a previous report, *DNMT1* physically interacted with *E2F1* in trophoblasts under hypoxic conditions (Figure 6H). Moreover, a ChIP assay further revealed that *DNMT1* knockdown increased *E2F1* enrichment in the *TP53BP2* promoter (Figure 6I). This result demonstrated that *DNMT1* mediates the binding between *E2F1* and the *TP53BP2* promoter. Collectively, these results suggest that *DNMT1* suppresses *E2F1* binding to the *TP53BP2* promoter, resulting in reduced autophagy in trophoblasts under hypoxic conditions.

### 3.7 G9a‐Mediated Histone Methylation Inhibits TP53BP2 Transcription in Trophoblasts

**FIGURE 7:**
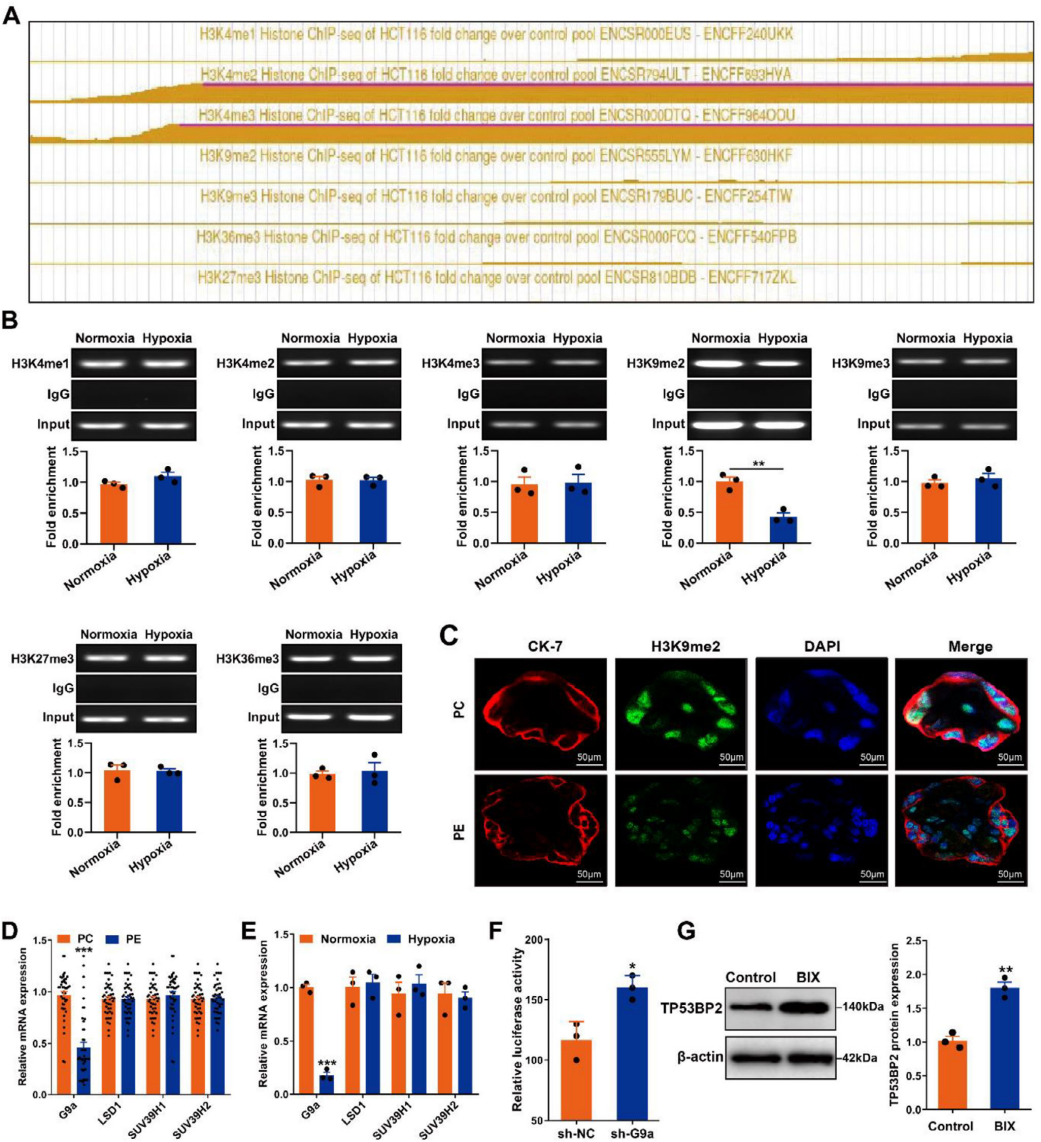
TP53BP2 is inhibited by G9a‐mediated histone methylation in trophoblasts. **(A)** *H3K4me1*, *H3K4me2*, *H3K4me3*, *H3K9me2*, *H3K9me3*, *H3K27me3* and *H3K36me3* modifications were detected in the *TP53BP2* promoter region. **(B)** ChIP assays were conducted to assess the enrichment of *H3K4me1*, *H3K4me2*, *H3K4me3*, *H3K9me2*, *H3K9me3*, *H3K27me3* and *H3K36me3* at the *TP53BP2* promoter in HTR8/SVneo cells under hypoxic conditions. **(C)** Immunofluorescence staining was used to detect the expression of *H3K9me2* (green) in placental trophoblasts. Scale bar = 50 µm. **(D, E)** The mRNA expression levels of *LSD1*, *SUV39H1*, *SUV39H2* and *G9a* were measured via qRT‒PCR in placentas and in HTR8/SVneo cells under hypoxic conditions. **(F)** Transcriptional activity of the *TP53BP2* promoter in HTR8/SVneo cells transfected with sh‐G9a under hypoxic conditions. **(G)** Expression of *TP53BP2* in HTR8/SVneo cells treated with BIX‐01294 (a G9a‐specific inhibitor) under hypoxic conditions was detected by western blotting. Data are presented as mean ± SD. Student's *t*‐test (unpaired, two‐tailed) was used to compare two independent groups. ^*^ *P*<0.05, ^**^ *P*<0.01, ^***^ *P*<0.001.

Histone modifications play an important role in regulating gene transcription [34]. This study used ENCODE Histone Modification Tracks embedded in the UCSC Genome Browser and identified seven histone modifications (*H3K4me1*, *H3K4me2*, *H3K4me3*, *H3K9me2*, *H3K9me3*, *H3K27me3* and *H3K36me3*) in the *TP53BP2* promoter region. Among these histone modifications, *H3K4me2* and *H3K4me3* showed the greatest number of enrichment peaks (Figure 7A). Using a ChIP assay, we detected a significant reduction in H3K9me2 enrichment at the *TP53BP2* promoter in HTR8/SVneo cells under hypoxic conditions, but not in other cells (Figure 7B). Immunofluorescence staining revealed a significant decrease in H3K9 me2 levels in placental trophoblasts from PE pregnancies (Figure 7C). This result confirms the importance of *H3K9me2* in *TP53BP2* transcription in trophoblasts of PE pregnancies. Furthermore, by measuring the levels of several widely recognized histone methyltransferases, including *G9a*, *LSD1*, *SUV39H1*, and *SUV39H2*, we detected a significant decrease in G9a levels in the placentas of PE pregnancies and HTR8/SVneo cells under hypoxic conditions (Figure 7D,E). Next, we investigated the effect of *G9a* on *TP53BP2* expression by establishing *G9a* knockdown in HTR8/Svneo cells. Among the three independent sh‐*G9a* constructs, sh‐*G9a* (#3) demonstrated the highest knockdown efficiency in HTR8/Svneo cells compared to sh‐ *G9a* (#1 and #2) (Figure S7). In vitro assays revealed that *G9a* knockdown in HTR8/Svneo cells significantly enhanced *TP53BP2* transcription under hypoxic conditions (Figure 7F). Similarly, treatment with BIX‐01294 (a *G9a*‐specific inhibitor) increased *TP53BP2* levels in HTR8/SVneo cells under hypoxic conditions (Figure 7G). These data demonstrate that *G9a* can inhibits *TP53BP2* expression in the placentas of patients with PE pregnancies.

### 3.8 G9a and DNMT1 Cooperatively Suppress E2F1 Binding to TP53BP2

**FIGURE 8:**
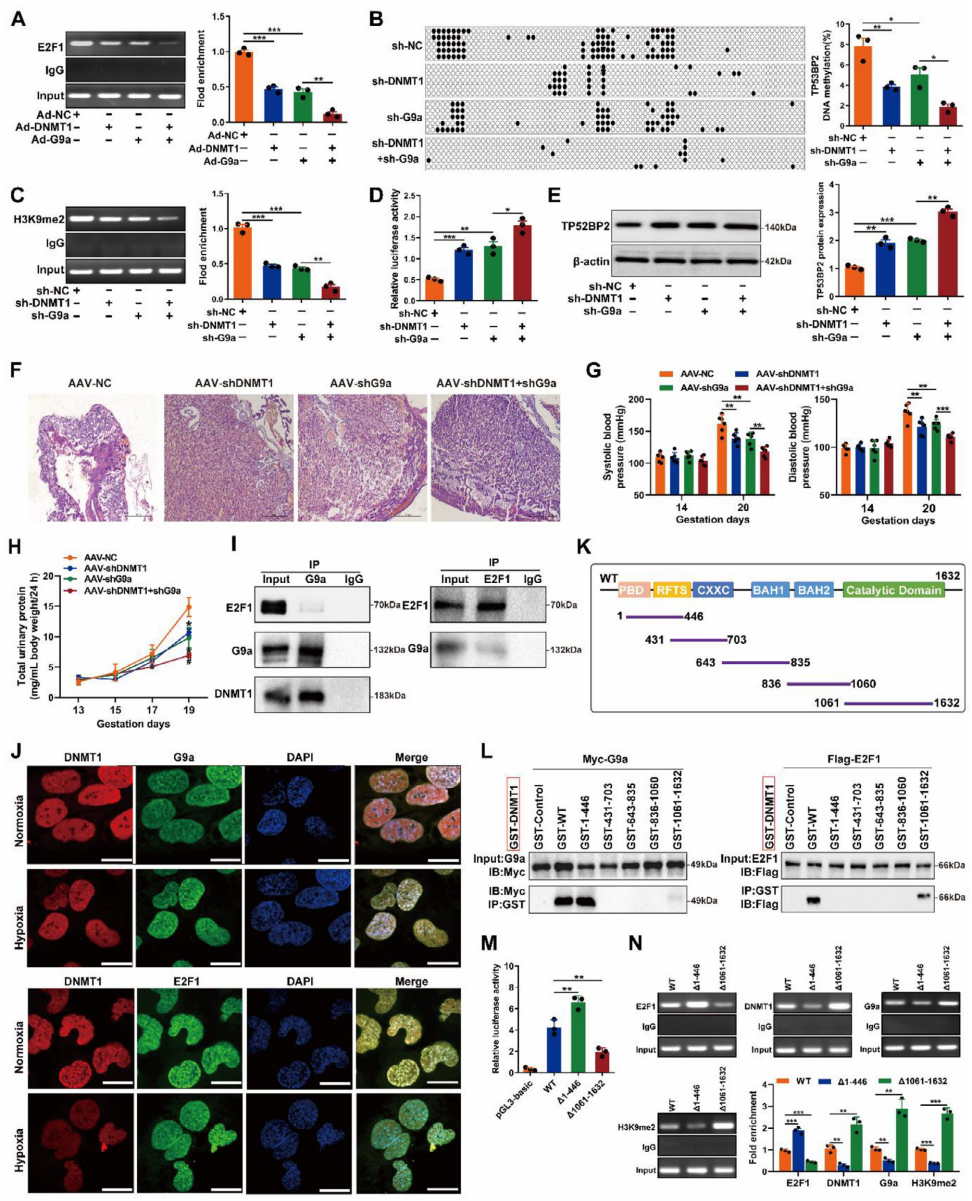
DNMT1 and G9a cooperatively regulate TP53BP2 expression in PE. **(A)** Enrichment of *E2F1* at the promoter region of *TP53BP2* was analyzed via ChIP in HTR8/SVneo cells transfected with Ad‐*DNMT1* and/or Ad‐*G9a* under hypoxic conditions. **(B)** BSP analysis was performed to determine the *TP53BP2* DNA methylation level in HTR8/SVneo cells transfected with sh‐*DNMT1* and/or sh‐*G9a* under hypoxic conditions. **(C)** *H3K9me2* enrichment at the promoter region of *TP53BP2* was analyzed via ChIP in HTR8/SVneo cells transfected with sh‐*DNMT1* and/or sh‐*G9a* under hypoxic conditions. **(D)** Promoter transcription activity of *TP53BP2* was analyzed using a dual‐luciferase reporter assay in HTR8/SVneo cells transfected with sh‐*DNMT1* and/or sh‐G9a under hypoxic conditions. **(E)** Western blotting was performed to determine *TP53BP2* expression in HTR8/SVneo cells transfected with sh‐*DNMT1* and/or sh‐*G9a* under hypoxic conditions. **(F)** Placental pathological changes in PE rats injected with AAV‐*shG9a* and/or AAV‐*shDNMT1* were evaluated using H&E staining. Scale bars = 500 µm. **(G)** Noninvasive tail‐cuff blood pressure measurement system used to detect the systolic blood pressure and diastolic blood pressure of PE rats injected with AAV‐*shG9a* and/or AAV‐*shDNMT1*. **(H)** Total urine protein levels in PE rats injected with AAV‐*shG9a* and/or AAV‐*shDNMT1*. **(I)** Co‐IP assay followed by immunoblotting showing the interactions between *G9a* and *DNMT1* or *E2F1* in HTR8/SVneo cells under hypoxic conditions. **(J)** Immunofluorescence staining was used to measure the colocalization of *DNMT1* (red) and *E2F1* (green) or *G9a* (green) in HTR8/SVneo cells under hypoxic conditions. The nuclei were stained with DAPI. Scale bar = 20 µm. **(K)** Schematic diagram depicting the structure of DNMT1 and truncation mutants of the GST‐tagged *DNMT1* fragments (GST‐Control, GST‐WT, GST‐1‐446, GST‐431‐703, GST‐643‐835, GST‐836‐1060, and GST‐1061‐1632). **(L)** The interactions between *DNMT1* and *G9a* or between *DNMT1* and *E2F1* were examined via Co‐IP with an anti‐Myc antibody in HEK293T cells cotransfected with plasmids encoding different GST‐tagged *DNMT1* fragments and plasmids encoding Myc‐tagged *G9a* (Myc‐*G9a*) or Flag‐tagged *E2F1* (Flag‐*E2F1*), respectively. **(M)** The transcriptional activity of the *TP53BP2* promoter in HTR8/SVneo cells transfected with the Δ1‐446 mutation or the Δ1061‐1632 mutation. **(N)** The enrichment of *E2F1*, *DNMT1*, *G9a* and *H3K9me2* at the promoter region of *TP53BP2* in HTR8/SVneo cells transfected with wild‐type *DNMT1* (WT), the G9a binding region deletion mutant *DNMT1* (Δ1‐446) or the E2F1 binding region deletion mutant *DNMT1* (Δ1061‐1632) was assessed via a ChIP assay under hypoxic conditions. Data are presented as mean±SD. Student's *t*‐test (unpaired, two‐tailed) was used to compare two independent groups, and a two‐way ANOVA test was performed for comparisons of multiple groups. ^*^ *P*<0.05, ^**^ *P*<0.01, ^***^ *P*<0.001; ^#^ *P*<0.05.

To elucidate the potential relationship between *DNMT1* and *G9a* in regulating *TP53BP2* expression, *DNMT1* or *G9a* was overexpressed in HTR8/SVneo cells via Ad‐DNMT1 and/or Ad‐G9a transfection (Figure S8). *DNMT1* or *G9a* expression decreased *E2F1* binding to the *TP53BP2* promoter, whereas co‐overexpression of *DNMT1* and *G9a* further attenuated this binding (Figure 8A). Moreover, *G9a* and *DNMT1* knockdown significantly decreased *TP53BP2* DNA methylation levels and H3K9me2 enrichment at the *TP53BP2* promoter in HTR8/SVneo cells under hypoxic conditions (Figure 8B,C), ultimately resulting in the significant upregulation of *TP53BP2* transcription and protein expression (Figure 8D,E). Similar results were observed in HTR8/SVneo cells treated with DC_05 and/or BIX under hypoxic conditions (Figure S9). Notably, AAV‐*shG9a* and/or AAV‐*shDNMT1* injection into the placenta of PE rats significantly increased placental damage, as evidenced by H&E staining (Figure 8F). Additionally, PE rats injected with AAV‐shG9a and/or AAV‐shDNMT1 showed significant increases in BP and urinary protein levels (Figure 8G,H), suggesting a synergistic inhibitory effect of *G9a* and *DNMT1* on *E2F1* binding to the *TP53BP2* promoter in placental dysfunction. Next, we investigated the interactions among *E2F1*, *DNMT1*, and *G9a* in trophoblasts. CoIP assays revealed that *DNMT1* physically interacted with *G9a* and *E2F1* in HTR8/SVneo cells under hypoxic conditions, whereas there was almost no interaction between G9a and E2F1 (Figure 8I). Immunofluorescence staining revealed that *DNMT1* and *G9a* were colocalized with *E2F1* in the nuclei of HTR8/SVneo cells (Figure 8J). Considering the multiple functional domains of *DNMT1*, a series of truncated constructs of DNMT1 were constructed, and plasmids encoding different GST‐tagged *DNMT1* fragments (GST‐control, GST‐WT, GST‐1‐446, GST‐431‐703, GST‐643‐835, GST‐836‐1060, and GST‐1061‐1632) were cotransfected with plasmids encoding Myc‐tagged *G9a* (Myc‐G9a) or Flag‐tagged *E2F1* (Flag‐E2F1) into HEK293 cells (Figure 8K). CoIP assays revealed that the 1–446 region of *DNMT1* interacted with G9a, whereas the 1061–1632 region of DNMT1 interacted with E2F1 (Figure 8L). Most importantly, a luciferase reporter assay revealed a marked increase in *TP53BP2* transcriptional activity in HTR8/SVneo cells transfected with the Δ1–446 mutation, whereas transfection with the Δ1061–1632 mutation decreased its transcriptional activity (Figure 8M). Furthermore, plasmids with a deleted regions of *DNMT1* that interact with *G9a* (Δ1–446) or E2F1 (Δ1061–1632) were constructed and transfected into HTR8/SVneo cells. Interestingly, deletion of the 1–446 region of *DNMT1* markedly increased *E2F1* enrichment at the *TP53BP2* promoter in HTR8/SVneo cells under hypoxic conditions while reducing *DNMT1* enrichment at the *TP53BP2* promoter. In contrast, the deletion of the 1061–1632 region of *DNMT1* promoted *E2F1* binding to the *TP53BP2* promoter and enhanced *H3K9me2* enrichment (Figure 8N). Collectively, these results indicate that the interaction between G9a and *DNMT1* suppresses the E2F1‐mediated activation of *TP53BP2* in trophoblasts.

## 4 Discussion

Preeclampsia (PE), a leading cause of maternal and fetal morbidity, is pathologically linked to placental dysfunction and abnormal trophoblast behavior [35, 36]. While previous studies have implicated autophagy in PE pathogenesis [37], the regulatory mechanisms governing aberrant autophagy in trophoblasts remain poorly understood. This study demonstrated that TP53BP2 could induce trophoblast autophagy in placentas from early‐onset PE pregnancies. Mechanistically, DNMT1‐mediated DNA hypomethylation and G9a‐mediated H3K9me2 suppressed E2F1 binding to the TP53BP2 promoter, eventually inhibiting TP53BP2 expression and autophagy in trophoblasts during PE pregnancies. Moreover, TP53BP2 is a predictive biomarker associated with the clinicopathological characteristics of early‐onset PE and a promising target for treating early‐onset PE.

TP53BP2, a core member of the ASPP family, is a critical determinant of cell fate through its physical interactions with p53‐family proteins and numerous signaling molecules. The observation that TP53BP2 is upregulated in PE placentas and hypoxia‐exposed trophoblasts, with its knockdown alleviating PE‐like pathology by inhibiting autophagy, extends our understanding of TP53BP2's pleiotropic roles. Beyond its established roles in apoptosis, TP53BP2 has emerged as a regulator of autophagy in diverse pathological contexts, mitigating organ injury and viral replication by modulating key autophagic processes [38, 39]. Our study identifies a novel pro‐autophagic role for TP53BP2 in trophoblasts, where it functions by activating Beclin‐1 through its release from the Beclin‐1‐Bcl‐2 complex. This tissue‐specific discrepancy highlights the context‐dependent nature of TP53BP2 signaling and underscores the need to investigate gene function within disease‐specific cellular microenvironments. Notably, this mechanism aligns with the established role of Beclin‐1‐Bcl‐2 interactions in balancing autophagy and survival [40], but our study is the first to link TP53BP2 to this pathway in trophoblasts, revealing a unique node in PE‐related autophagic dysregulation.

The epigenetic regulation of gene expression involves several mechanisms, including DNA methylation, histone modification, and biogenesis and action of noncoding RNAs [41], which regulate gene expression by modulating the accessibility of transcription factors and other regulatory proteins to DNA. Pregnancy involves dynamic genetic and epigenetic modifications essential for the development and health of the mother and the fetus. These modifications encompass a myriad of processes that influence gene expression, chromatin structure, and cellular function throughout gestation [42], but how coordinated DNA methylation and histone modification regulate key functional genes in PE remains unclear. Our findings that TP53BP2 upregulation is driven by DNMT1‐mediated DNA hypomethylation and G9a‐dependent H3K9me2 reduction represent a significant advance. Under physiological conditions, DNMT1 maintains DNA methylation, while G9a catalyzes the repressive H3K9me2 mark at the promoter. Critically, we demonstrate that these two mechanisms do not operate in isolation. The physical interaction between G9a and the N‐terminal domain (amino acids 1–446) of DNMT1 suggests a mechanism for their co‐recruitment and cooperative action. This DNMT1‐G9a complex fosters a repressive chromatin environment: H3K9me2 provides a docking platform that reinforces DNMT1 recruitment and activity, leading to sustained DNA methylation, thereby repressing TP53BP2 expression in PE.

E2F1, a transcription factor traditionally associated with cell cycle control and oncogenesis [43, 44], has recently emerged as a regulator of autophagy [45, 46]. Our findings confirmed that E2F1 is upregulated in PE placentas and hypoxic trophoblasts, and its elevated level is both necessary and sufficient to drive TP53BP2 transcription and subsequent autophagy. This is mechanistically supported by the identification of three functional E2F1 binding sites within the TP53BP2 promoter, directly linking E2F1 activity to the pro‐autophagic gene program in trophoblasts. The pivotal question, therefore, shifts to understanding how E2F1's access to the TP53BP2 promoter is controlled. We discovered that this access is restrictively gated by a synergistic epigenetic barrier erected by DNMT1 and G9a. The compact, transcriptionally silent chromatin state formed by their cooperative action sterically hinders E2F1 from accessing its cognate binding sites. In PE, the upregulation of DNMT1 and G9a disrupts this cooperative repression. The increase in DNA methylation and H3K9me2 marks reinforces the epigenetic barrier, converting the TP53BP2 promoter from an open to a closed chromatin state. This chromatin remodeling serves as a key event that prevents E2F1 binding and suppresses TP53BP2 transcription in hypoxic trophoblasts. Therefore, the pathological upregulation of TP53BP2 results not merely from low E2F1 levels, but more precisely from hypoxia‐induced DNMT1/G9a‐mediated epigenetic mechanisms that lock the promoter and suppress E2F1‐driven transcription.

## 5 Conclusion

**FIGURE 9:**
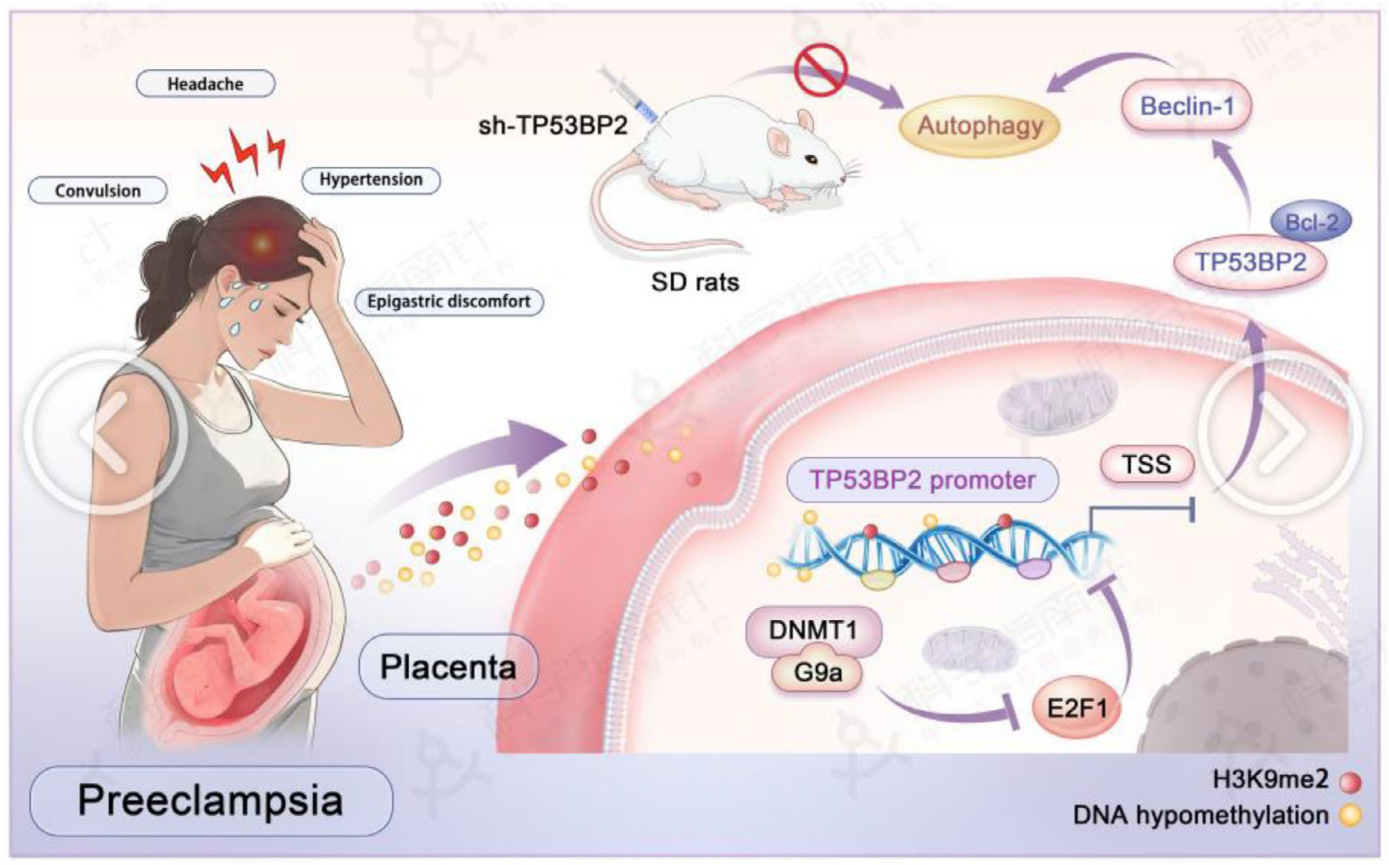
TP53BP2 promotes placental autophagy and preeclampsia via G9a, and DNMT1 cooperatively modulates E2F1. The inhibition of *TP53BP2* expression attenuates the progression of PE by inhibiting the autophagy of trophoblasts in SD rats; this is attributed to the fact that G9a‐mediated H3K9me2‐ and *DNMT1*‐mediated DNA hypomethylation suppressed the binding of *E2F1* at the *TP53BP2* promoter to suppress the expression of *TP53BP2* transcription and subsequently inhibited the release of Beclin‐1 from the Bcl‐2‐Beclin‐1 complex.

This study has several limitations that should be addressed in future work. A primary limitation is the reliance on a single trophoblast cell line (HTR8/SVneo) for the in vitro experiments; utilizing primary trophoblast cultures or organoid models would more faithfully mimic the in vivo placental microenvironment. Additionally, the clinical utility of TP53BP2 as a predictive biomarker requires validation in larger, multicenter cohorts with long‐term follow‐up of maternal and fetal outcomes. In summary, this study delineates a novel epigenetic pathway‐the DNMT1/G9a‐E2F1‐TP53BP2 axis‐that promotes aberrant trophoblast autophagy in early onset preeclampsia (Figure 9). By establishing TP53BP2 as a critical driver of placental dysfunction and a promising therapeutic target, our work offers a conceptual advance in understanding PE pathogenesis. Future studies addressing the current limitations will be essential to translate these findings into targeted diagnostic and therapeutic strategies for early onset of PE.

## Conflicts of Interest

The authors declare no conflict of interests.

## Supporting information




**Supporting File 1**: advs73524‐sup‐0001‐SuppMat.docx.

**Supporting File 2**: advs73524‐sup‐0002‐TableS1‐S3.docx.

## Data Availability

The data that support the findings of this study are available on request from the corresponding author. The data are not publicly available due to privacy or ethical restrictions.
