# Supplementary material for: TP53BP2 Promotes Placental Autophagy and Preeclampsia via G9a and DNMT1 Cooperatively Modulating E2F1
Source: Adv Sci (Weinh). 2026 Jan 7;13(10):e16408. doi: 10.1002/advs.202516408 (PMC12915100; doi:10.1002/advs.202516408)
Supplement: Supplementary file 1 — Supporting File 1: advs73524‐sup‐0001‐SuppMat.docx. [file ADVS-13-e16408-s002.docx]

Supporting Information

*TP53BP2* Promotes Placental Autophagy and Preeclampsia via G9a and *DNMT1* Cooperatively Modulating E2F1

**Nan Jiang^1^**^†^**, Shaoju Jin^2^**^†^**, Shaoying Wen^3,4^, Wen Zeng^5^, Chen Wang^3,4^，Jingyu Wang^6^, Qingyun Song^7^, Guizhong Li^3,4^, Pengzhi Yin^8^, Yuhui Liao^9*^, Yuee Chai^10*^, Huiping Zhang^3,4,11*^, Shengchao Ma^3,4*^**

**^1^** School of Basic Medical Science, Central South University, Changsha, 410078, China.

**^2^** LuoHe Medical College, HeNan province, LuoHe,462000, China

**^3^** NHC Key Laboratory of Metabolic Cardiovascular Diseases Research, Ningxia Medical University, Yinchuan, 750004, China.

**^4^** Key Laboratory of Vascular Injury and Repair Research of the Ningxia the Ningxia Hui Autonomous Region, Ningxia Medical University, Yinchuan ,750004, China

**^5^** Department of Scientific Research and Teaching, the Central Hospital of Shaoyang City, Shaoyang, 422000, China

**^6^** Xiangya Medical College, Central South University, Changsha, 410078, China

**^7^** College of Life Sciences, Central South University, Changsha, 410078, China

**^8^** Faculty of Biomedical Engineering, the Chinese University of Hong Kong, Hong Kong, China

**^9^** Institute for Engineering Medicine, Kunming Medical University, Kunming, 650500, China

**^10^** State Key Laboratory of Functions and Applications of Medicinal Plants, Guizhou Provincial Engineering Technology Research Center for Chemical Drug R&D, Guiyang, 550004, China.

**^11^** Department of Medical Genetics, Maternal and Child Health Hospital of Hunan Province, Changsha,410008, China

† These authors contributed equally to this work.

**^*^Correspondence**: Shengchao Ma (solarmsc@163.com); Huiping Zhang(zhp19780620@163.com);Yuee Chai(caiyuee@gmc.edu.cn); Yuhui Liao(liaoyh8@mail.sysu.edu.cn)

**
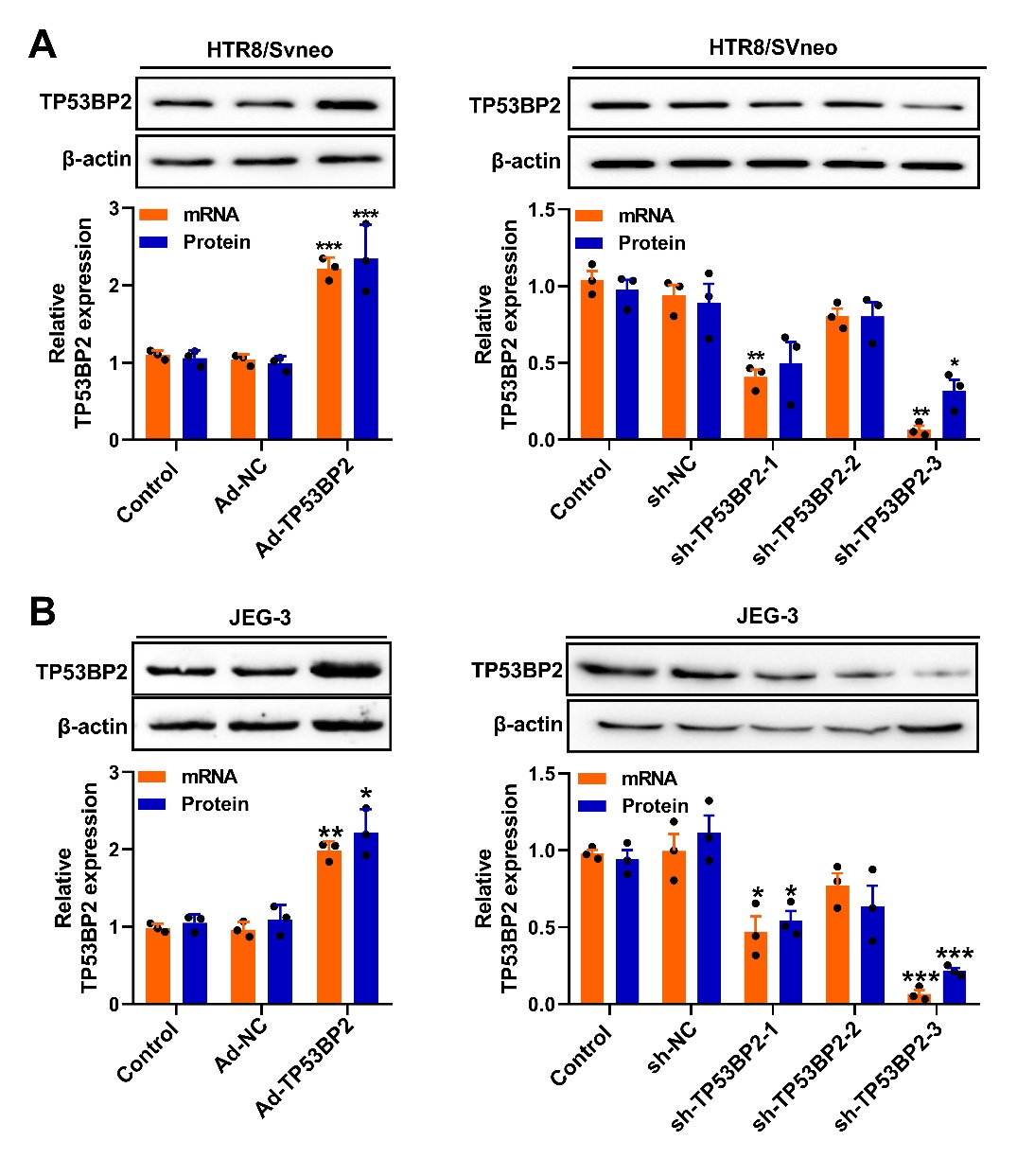
**

**Fig****ure S1. Expression of TP53BP2 in HTR8/SVneo and JEG-3 cells. (A, B)** The expression of TP53BP2 in HTR8/SVneo and JEG-3 cells transfected with Ad-TP53BP2 or sh-TP53BP2 was examined via qRT‒PCR and western blotting. The data are presented as the means ± SDs. ^*^*P*<0.05, ^**^*P*<0.01, ^***^*P*<0.001.


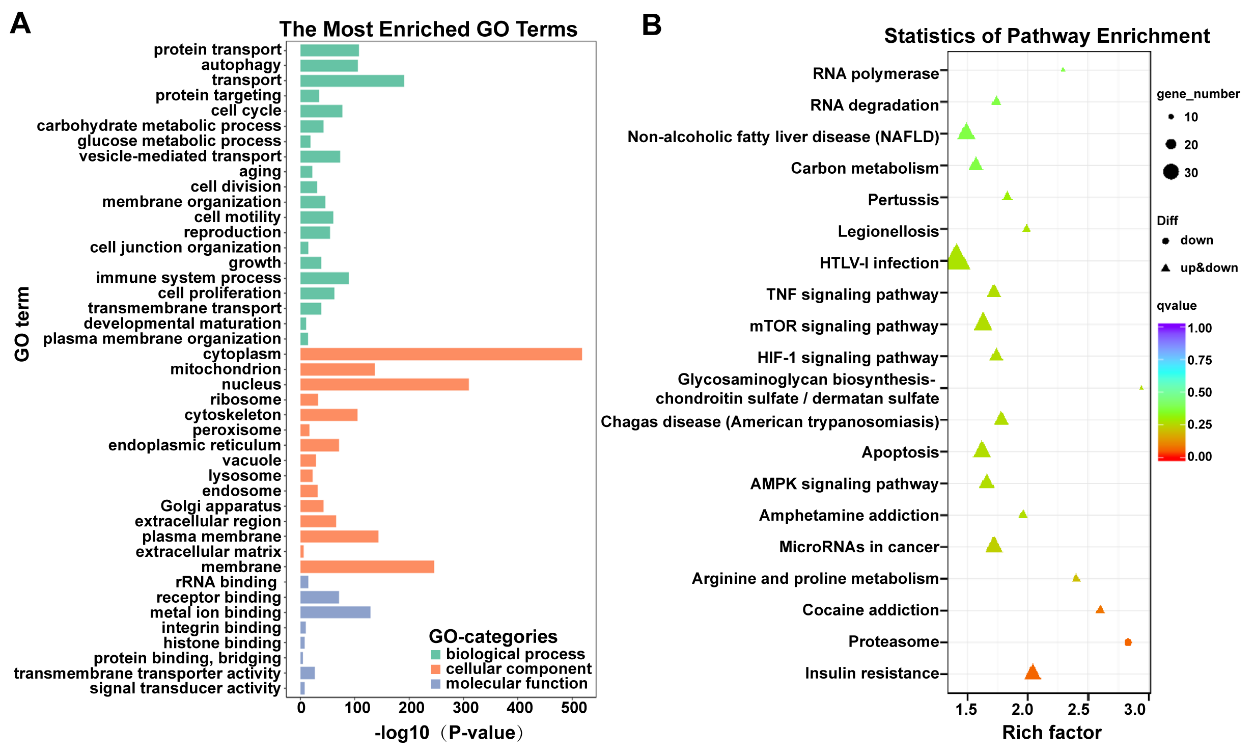


**Figure S2.** **GO and KEGG enrichment analyses of DEGs in trophoblasts transfected with sh-TP53BP2. (A)** Histogram of the results of the Gene Ontology (GO) enrichment analysis of the DEGs. The GO terms included biological processes, molecular functions, and cellular components. The vertical axis represents the GO annotation; the horizontal axis represents the number of genes. **(B)** Scatter plot of the results of the KEGG enrichment analysis of DEGs.

**
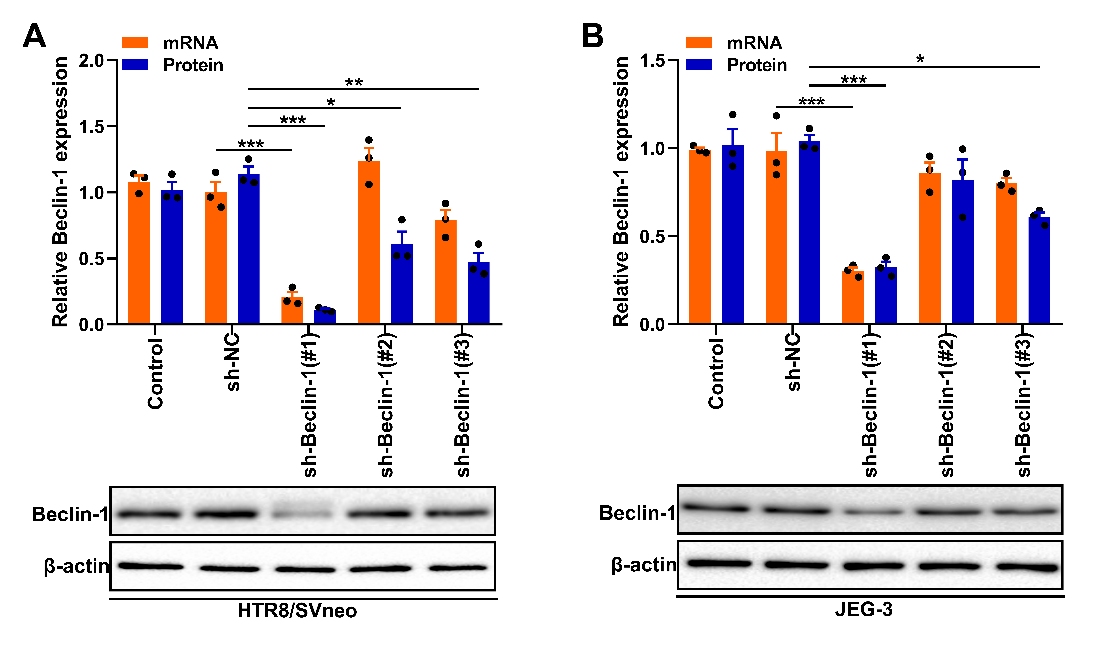
**

**Figure S3. Expression of Beclin-1 in HTR8/SVneo and JEG-3 cells. (A, B)** The expression of Beclin-1 in HTR8/SVneo and JEG-3 cells transfected with three sh-Beclin-1 or sh-NC strains was examined via qRT‒PCR and western blotting. The data are presented as the means ± SDs. ^*^*P*<0.05, ^**^*P*<0.01, ^***^*P*<0.001.

**
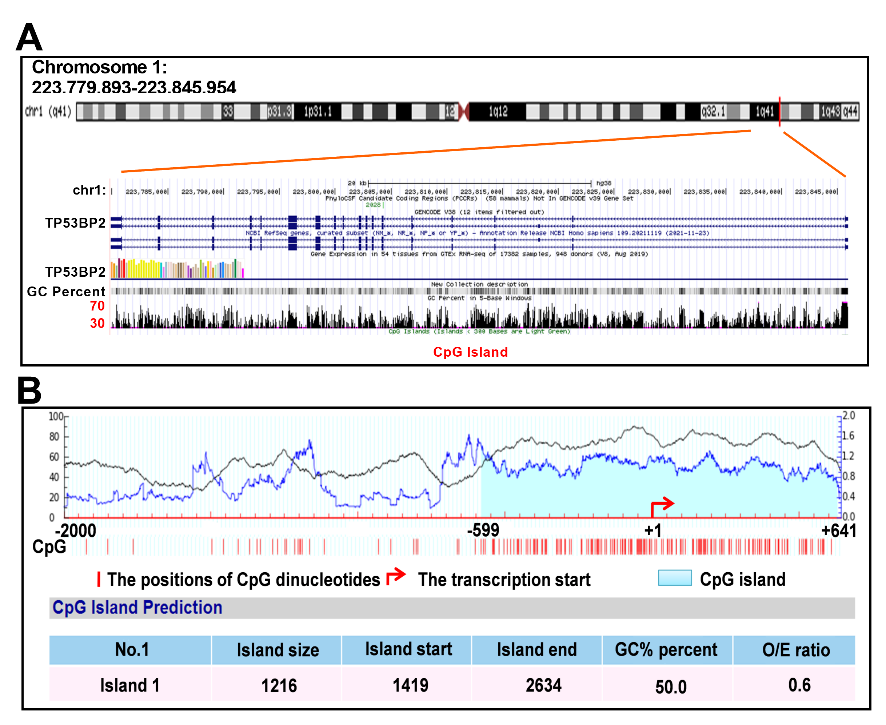
**

**Figure S4. TP53BP2 transcript analysis. (A)** The UCSC genome browser (http://genome.ucsc.edu) was used to visualize the genomic positioning and organization of TP53BP2, which is located on chromosome 1q41. The genomic region spanning approximately 60 kb is displayed, and gene transcripts are shown in the tracks below the genomic regions. Additionally, the percentages of CpG islands and GCs are shown in the region below the gene transcript. **(B)** The Meth primer program identified one CpG island of 1240 bp (-599/+641 bp) at the TP53BP2 promoter. The red arrow indicates the transcription start site. The lower limits were as follows: %GC=50, obsCpG/exp CpG=0.60, and length=200 bp. CpG island star=1419, end=2634, %GC=74.4, obsCpG/expCpG=0.944.


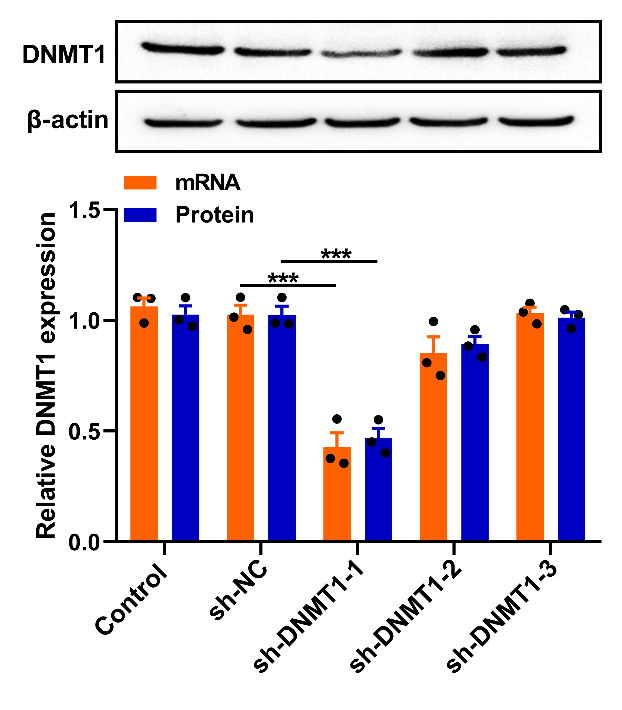


**Figure S5. Expression of** **DNMT1 in HTR8/SVneo cells.** The expression of DNMT1 was examined by qRT‒PCR and western blot in HTR8/SVneo cells transfected with three sh-DNMT1 or sh-NC. Data were presented as the mean ± SD. ^***^*P*<0.001.

**
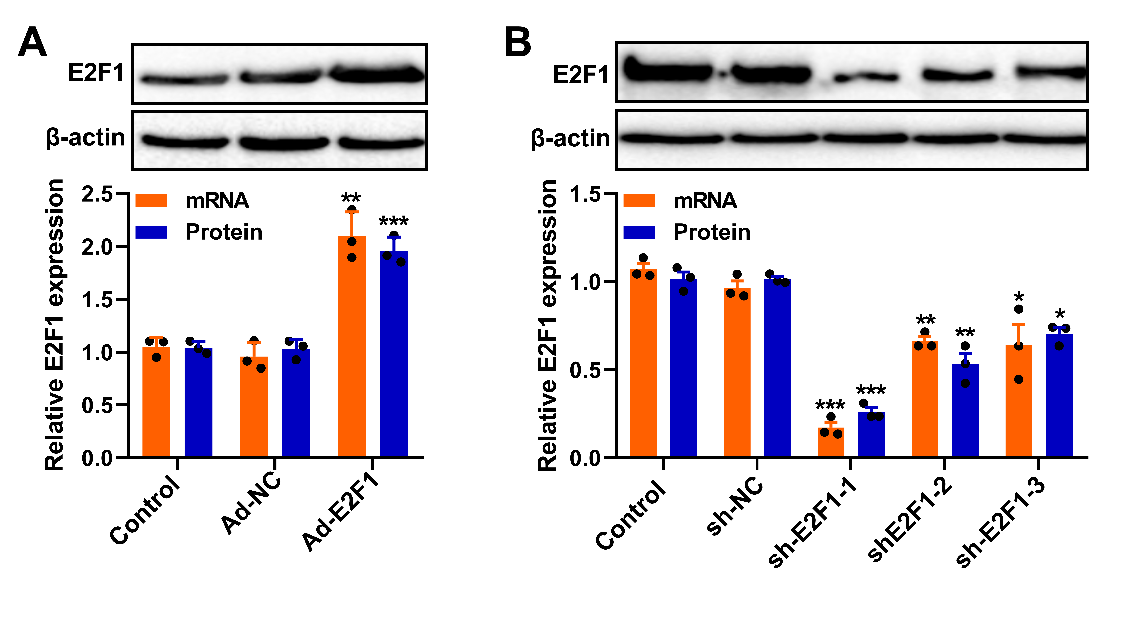
**

**Figure S6. Expression of E2F1 in HTR8/SVneo cells. (A, B)** The expression of ASPP2 in HTR8/SVneo cells transfected with Ad-E2F1 or sh-E2F1 was examined via qRT‒PCR and western blotting. The data are presented as the means ± SDs. ^*^*P*<0.05, ^**^*P*<0.01, ^***^*P*<0.001.


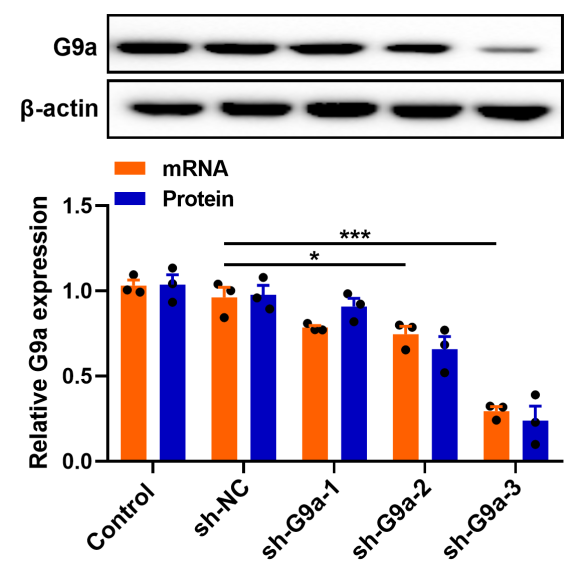


**Figure S7. Expression of G9a in HTR8/Svneo cells.** The expression of G9a in HTR8/SVneo cells transfected with sh-G9a or sh-NC was examined via qRT‒PCR and western blotting. The data are presented as the means ± SDs. ^*^*P*<0.05, ^***^*P*<0.001.


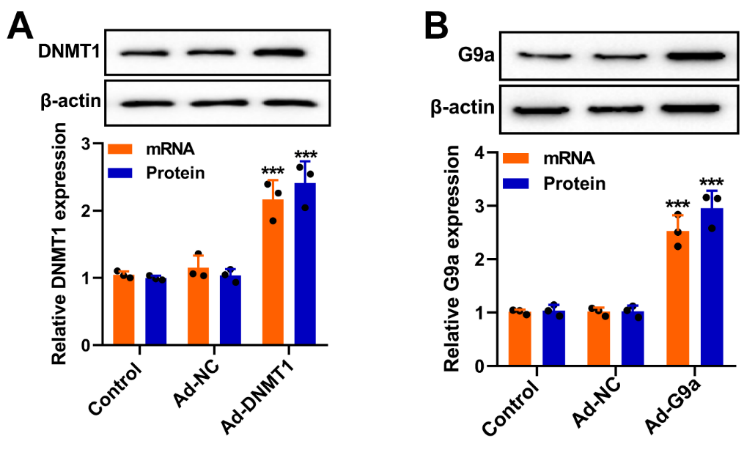


**Figure S8. Expression of DNMT1 and G9a in HTR8/Svneo cells. (A, B)** The expression of DNMT1 and G9a was examined by qRT‒PCR and western blot in HTR8/SVneo cells transfected with Ad-DNMT1 or Ad-G9a. Data were presented as the mean ± SD. Student’s t test (unpaired, two-tailed) was used to compare two independent groups. ^***^*P*<0.001.


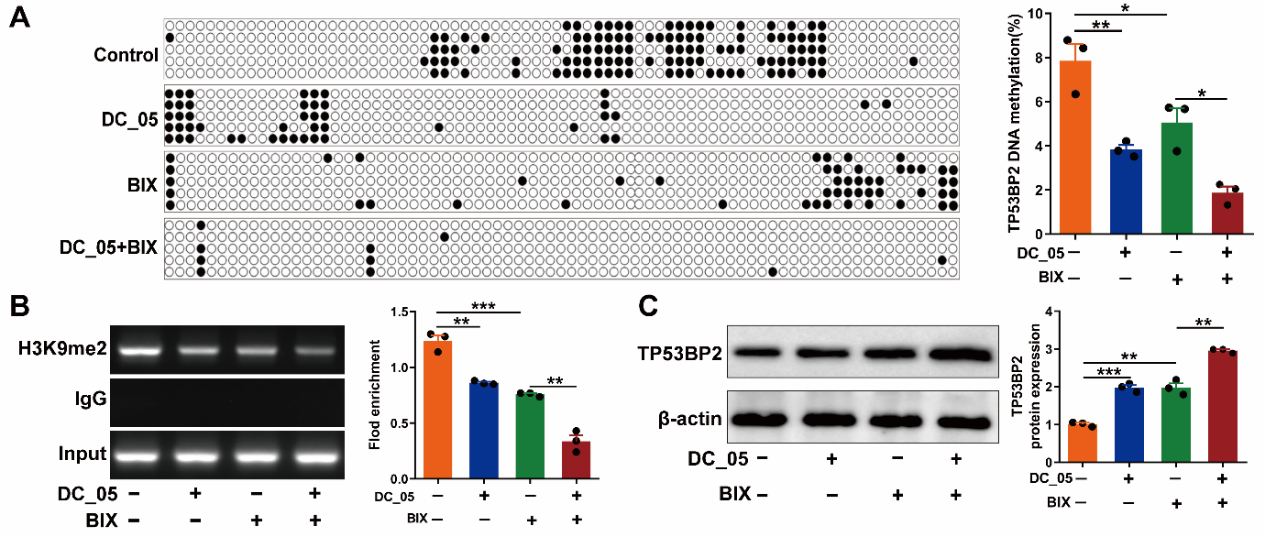


**Figure S9. Effects of DC_05 and BIX on TP53BP2 DNA methylation, H3K9me2 and H3K9me2 expression in HTR8/Svneo cells. (A)** BSP analysis was performed to determine the TP53BP2 DNA methylation level in HTR8/SVneo cells under hypoxia. **(B)** H3K9me2 enrichment at the TP53BP2 promoter in HTR8/SVneo cells under hypoxia was assessed via a ChIP assay. **(C)** TP53BP2 expression in HTR8/SVneo cells under hypoxia was determined by western blotting. The data are presented as the means ± SDs. ^*^*P*<0.05, ^**^*P*<0.01, ^***^*P*<0.001.
