# Supplementary material for: TP53BP2 Promotes Placental Autophagy and Preeclampsia via G9a and DNMT1 Cooperatively Modulating E2F1
Source: Adv Sci (Weinh). 2026 Jan 7;13(10):e16408. doi: 10.1002/advs.202516408 (PMC12915100; doi:10.1002/advs.202516408)
Supplement: Supplementary file 2 — Supporting File 2: advs73524‐sup‐0002‐TableS1‐S3.docx. [file ADVS-13-e16408-s001.docx]

**Supplementary Tables**

*TP53BP2* Promotes Placental Autophagy and Preeclampsia via G9a and *DNMT1* Cooperatively Modulating E2F1

**Nan Jiang^1^**^†^**, Shaojv Jin^2^**^†^**, Shaoying Wen^3,4^, Wen Zeng^5^, Chen Wang^3,4^，Jingyu Wang^6^, Qingyun Song^7^, Guizhong Li^3,4^, Pengzhi Yin^8^, Yuhui Liao^9*^, Yuee Chai^10*^, Huiping Zhang^3,4,11*^, Shengchao Ma^3,4*^**

**^1^** School of Basic Medical Science, Central South University, Changsha, 410078, China.

**^2^** LuoHe Medical College, HeNan province, LuoHe,462000, China

**^3^** NHC Key Laboratory of Metabolic Cardiovascular Diseases Research, Ningxia Medical University, Yinchuan, 750004, China.

**^4^** Key Laboratory of Vascular Injury and Repair Research of the Ningxia the Ningxia Hui Autonomous Region, Ningxia Medical University,Yinchuan ,750004, China

**^5^** Department of Scientific Research and Teaching, the Central Hospital of Shaoyang City, Shaoyang, 422000, China

**^6^** Xiangya Medical College, Central South University, Changsha, 410078, China

**^7^** College of Life Sciences, Central South University, Changsha, 410078, China

**^8^** Faculty of Biomedical Engineering, the Chinese University of Hong Kong, Hong Kong, China

**^9^** Institute for Engineering Medicine, Kunming Medical University, Kunming, 650500, China

**^10^** State Key Laboratory of Functions and Applications of Medicinal Plants, Guizhou Provincial Engineering Technology Research Center for Chemical Drug R&D, Guiyang, 550004 , China.

**^11^** Department of Medical Genetics, Maternal and Child Health Hospital of Hunan Province, Changsha,410008, China

† These authors contributed equally to this work.

**^*^Correspondence:** Shengchao Ma (solarmsc@163.com); Huiping Zhang(zhp19780620@163.com);Yuee Chai(caiyuee@gmc.edu.cn); Yuhui Liao(liaoyh8@mail.sysu.edu.cn)

**Table S1. The primer sequences for qRT-PCR analysis**

| Gene | Species | Primer sequence, 5' to 3' |
| --- | --- | --- |
| TP53BP2 | Human | Forward: 5’- ATTGAATCAAGAGCAGAATGCC-3’  Reverse: 5’- CAGCTCATTAACACGCTTATCC-3’ |
| G9a | Human | Forward: 5’-ATGGTAAGGTCGGTGTGA-3’  Reverse: 5’-AATCTCCACTTTGCCACTGC-3’ |
| E2F1 | Human | Forward: 5’-GATTATAAATCTAGCCTGATTC-3’  Reverse: 5’-TTGTGTTCTTCTTTACCCTC-3’ |
| SUV39H1 | Human | Forward: 5’- CCTGCCCTCGGTATCTCTAAG-3’  Reverse: 5’- ATATCCACGCCATTTCACCAG-3’ |
| SUV39H2 | Human | Forward: 5’- TCTATGACAACAAGGGAATCACG-3’  Reverse: 5’- GAGACACATTGCCGTATCGAG-3’ |
| LSD1 | Human | Forward: 5’- AATCAGAGTACATGCGACTGAGA-3’  Reverse: 5’- GCTGTATCCTTCGCTGTTTCC-3’ |
| GAPDH | Human | Forward: 5’-CATGTACGTTGCTATCCAGGC-3’  Reverse: 5’-CTCCTTAATGTCACGCACGAT-3’ |

**Table S2. Primer sequences for MSP analysis**

| Primer set | Primer sequence, 5' to 3' |
| --- | --- |
| TP53BP2-O | Forward: 5′-GGTGCCATTCACTACCATCC-3′  Reverse: 5′-GGCCGCCTTTTCTTTCTTC-3′ |
| TP53BP2-M | Forward: 5′-GGTGCCATTCACTACCATCC-3′  Reverse: 5′-CCGCTTCTTTCTTTTCCGC-3′ |
| TP53BP2-U | Forward: 5′-CTCGGTGCCATTCACTACCA-3′  Reverse: 5′-CCGCTTCTTTCTTTTCCGC-3′ |

O, outer primer; M, methylation primer; U, unmethylation primer.

**Table S3. The primer sequences for ChIP-PCR analysis**

| The binding sites of E2F1  at TP53BP2 promoter | Primer sequence, 5' to 3' |  |
| --- | --- | --- |
| TP53BP2 (-33/-22) | Forward: 5’-GCCATTCACTACCATCCTGTT-3’ |  |
|  | Reverse: 5’-GGCCGCCTTTTCTTTCTTC-3’ | |
| TP53BP2 (-99/-88) | Forward: 5’-GGTGCCATTCACTACCATCC-3’ |  |
|  | Reverse: 5’-CTTTCTTCGCCCCGGACT-3’ | |
| TP53BP2 (-368/-357) | Forward: 5’-CCCTGTTCTGAAGGCAAAGG-3’ |  |
|  | Reverse: 5’-TCAGGAAAACAGGATGGTAGTG-3’ | |
